# Plasmid-Encoded RepA Proteins Specifically Autorepress Individual *repABC* Operons in the Multipartite *Rhizobium leguminosarum* bv. *trifolii* Genome

**DOI:** 10.1371/journal.pone.0131907

**Published:** 2015-07-06

**Authors:** Kamil Żebracki, Piotr Koper, Małgorzata Marczak, Anna Skorupska, Andrzej Mazur

**Affiliations:** Department of Genetics and Microbiology, Institute of Microbiology and Biotechnology, Faculty of Biology and Biotechnology, Maria Curie-Skłodowska University, Lublin, Poland; University of Manchester, UNITED KINGDOM

## Abstract

Rhizobia commonly have very complex genomes with a chromosome and several large plasmids that possess genes belonging to the *repABC* family. RepA and RepB are members of the ParA and ParB families of partitioning proteins, respectively, whereas RepC is crucial for plasmid replication. In the *repABC* replicons, partitioning and replication functions are transcriptionally linked resulting in complex regulation of *rep* gene expression. The genome of *R*. *leguminosarum* bv. *trifolii* TA1 (RtTA1) consists of a chromosome and four plasmids (pRleTA1a-d), equipped with functional *repABC* genes. In this work, the regulation of transcription of the individual *repABC* cassettes of the four RtTA1 plasmids was studied. The involvement of the RepA and RepB as well as *parS*-like centromere sites in this process was depicted, demonstrating some dissimilarity in expression of respective *rep* regions. RtTA1 *repABC* genes of individual plasmids formed operons, which were negatively regulated by RepA and RepB. Individual RepA were able to bind to DNA without added nucleotides, but in the presence of ADP, bound specifically to their own operator sequences containing imperfect palindromes, and caused operon autorepression, whereas the addition of ATP stimulated non-specific binding of RepA to DNA. The RepA proteins were able to dimerize/oligomerize: in general dimers formed independently of ATP or ADP, although ATP diminished the concentration of oligomers that were produced. By the comprehensive approach focusing on a set of plasmids instead of individual replicons, the work highlighted subtle differences between the organization and regulation of particular *rep* operons as well as the structures and specificity of RepA proteins, which contribute to the fine-tuned coexistence of several replicons with similar *repABC* cassettes in the complex bacterial genome.

## Introduction

Alphaproteobacteria commonly have very complex genomes with a chromosome and plasmids, which may significantly vary in the size and content. Bacteria of the *Rhizobiaceae* family have numerous, large plasmids, all belonging to the *repABC* family [1]. An important feature of rhizobial plasmids is their low-copy number in the cell and such plasmids rely on partitioning systems (*par*), distributing newly replicated plasmids to daughter cells [2–4]. Partitioning systems have been well described for several enterobacterial strains harbouring single plasmids like R or F, and episomal prophages like P1 [5–7]. Most of the low-copy number plasmids possess a Type I segregation system with Walker-type ATPase (MinD/ParA superfamily) [1]. Subtype Ia comprises large ATPases with a DNA-binding domain in their N-terminus [8, 9].

The *repABC* plasmid family has several characteristics in common. Most notably, all elements necessary for active segregation and replication, as well as those responsible for incompatibility, are located in the same operon [10, 11], and the genetic organization of the *repABC* cassette is well conserved: *repA* is upstream of *repB*, and both precede *repC* [1, 11–13]. Despite their apparent structural homogeneity, *repABC* operons have diverse DNA sequences. They vary with respect to the presence of peptide-encoding minigenes, the numbers and class of the regulatory elements involved in operon transcription, and the numbers and positions of centromeric *parS* sequences [1, 14]. The structural diversity of *repABC* operons resulting from their complex and independent evolutionary history [15] may affect the regulation and functioning of particular replication systems.

RepA and RepB are members of the ParA and ParB families of partitioning proteins, respectively [6]. The size and sequence similarities of the RepA and RepB suggest that the partition systems of most of the rhizobial *repABC* replicons are Type Ia [1]. RepA and RepB play dual roles in plasmid maintenance: in conjunction with *parS* centromere-like sites, they participate in partitioning and in the negative transcriptional regulation of their own operons [16–20]. RepA-ADP can bind specifically to the operator preceding the *repABC* operon of p42d (formally known as pRetCFN42d) for repression of transcription [21]. RepC protein is crucial for plasmid replication and does not show similarities with members of other protein families. Its expression, down-regulated by small antisense RNA, encoded in the *repB-repC* intergenic region [22–27], is required and sufficient for replication of a plasmid [10, 28], which indicates that the origin of replication lies within the *repC* gene [28, 29]. Comprehensive analyses of the mechanisms of replication, segregation, and transcriptional regulation of several *repABC* operons coexisting in one cell are still scarce especially for the rhizobial model.

The genome of *R*. *leguminosarum* bv. *trifolii* TA1 (RtTA1) consists of five replicons: the chromosome and four plasmids pRleTA1a, pRleTA1b, pRleTA1c and pRleTA1d (497 kb, 598 kb, 646 kb and 798 kb, respectively), with the smallest, pRleTA1a, recognized as a symbiotic plasmid (pSym) carrying genes responsible for symbiosis with clover [30]. Recently, we have shown that all RtTA1 plasmids are equipped with functional *repABC* genes [31]. The complex evolutionary history and structural diversity of the RtTA1 *repABC* cassettes was demonstrated. They differed in the numbers and positions of *parS* and incompatibility elements (*incα*) located in the *repB-repC* intergenic regions, and this was especially apparent in the case of pSym. Incompatibility assays with recombinant constructs containing *parS* or *incα* demonstrated that RtTA1 plasmids belong to different incompatibility groups and were acquired by separate events of lateral transfer, as shown by phylogenetic analyses. pRleTA1a (pSym) was probably the evolutionary newest plasmid of this strain [31].

The aim of this study was to provide molecular characterization of partition proteins of four plasmids coexisting in one cell. Special attention was paid to the domain structure, and specific DNA-binding properties of RepA proteins of individual RtTA1 plasmids. The regulation of transcription of four *repABC* cassettes was studied in detail and the involvement of the RepA and RepB as well as *parS*-like elements in this process was depicted, demonstrating some dissimilarity in the expression of the *rep* cassettes of respective plasmids. Operators constituting RepA binding sites of the individual RtTA1 plasmids were mapped. Moreover, the oligomerization propensity of the individual RepA proteins was analysed and its role in both the plasmid partition and regulation of transcription of *repABC* genes was discussed. To our knowledge, this is the first successful attempt at such a comprehensive approach focused on a set of plasmids instead of individual ones that sheds light on the problem of coexistence of several replicons equipped with similar *repABC* cassettes in the complex bacterial genome.

## Materials and Methods

### Bacterial strains and growth conditions

The bacterial strains and plasmids used in this work are listed in Table 1. *Escherichia coli* strains were cultured at 37°C or 30°C (*E*. *coli cya* strain DHM1) in LB medium [32]. *Rhizobium* and *Agrobacterium* strains were grown at 28°C in 79CA medium [33] or in M1 minimal medium [32]. Antibiotics, when used, were added at the following final concentrations (μg/ml): ampicillin, 100; kanamycin, 40 (25 for propagation of *E*. *coli* M15[pREP4] strain); rifampicin, 40; chloramphenicol, 30; tetracycline, 40; gentamicin, 10 for *Rhizobium*, 5 for *E*. *coli*.

**Table 1:** Strains and plasmids used in this study.

Strains and plasmids	Description	Source or reference
*E*. *coli*		
DH5α	*supE*44 Δ*lac*U169 (θ80 *lacZ*ΔM15) *hsdR*17 *recA*1 *endA*1 *gyrA*96 *thi*-1 *relA*1	[32]
JM101	*supE thi-1* Δ(*lac*-*proAB*) F′ [*traD36 proAB* ^+^ *lacI* ^q^ *lacZ*Δ*M15*]	[32]
M15[pREP4]	Nal^S^, Str^S^, Rif^S^, Thi^-^, Lac^-^, Ara^+^, Gal^+^, Mtl^-^, F^-^, RecA^+^, Uvr^+^, Lon^+^, carrying repressor vector pREP4	Qiagen
DHM1	F^-^, *cya-854*, *recA1*, *endA1*, *gyrA96 (Nal* ^*r*^ *)*, *thi1*, *hsdR17*, *spoT1*, *rfbD1*, *glnV44(AS)*	[42]
Rosetta 2(DE3)pLysS	F^-^ *ompT hsdS*_B_(r_B_^-^m_B_^-^) *gal dcm* (DE3) pLysSpRARE2 (Cm^r^)	Novagen
*Rhizobium*		
TA1	*R*. *leguminosarum* bv. *trifolii*, wild type, Str^r^, Rif^r^	[50]
*Agrobacterium*		
GMI9023	*A*. *tumefaciens* Str^r^, Rif^r^ cured of pAt and pTi	[51]
*Plasmids*		
pBBR1MCS-2	*mob*, Km^r^	[35]
pBBR1MCS-5	*mob*, Gm^r^	[35]
pQE-30	*ori* ColE1, Amp^r^, expression vector	Qiagen
pET-32a(+)	*ori* pBR322, Amp^r^, *trxA*, expression vector	Novagen
pMP220	IncP, *mob*, promoterless *lacZ*, Tet^r^	[34]
pMPK	pMP220 derivative with Kan^r^ cassette from pBBR1MCS-2 inserted in *Eco*RI-*Kpn*I sites	This work
pMPK derivatives—*lacZ* transcriptional fusions		
*repABC* of pRleTA1a		
pMa1-pMa3	pMPK with 576 bp, 269 bp, 198 bp *Kpn*I-*Xba*I fragments comprising respectively 433 bp, 126 bp and 55 bp upstream of *repA* and 143 bp of *repA*	This work
pMa/ABC2	pMPK with 2956 bp *Kpn*I-*Xba*I fragment comprising 269 bp upstream of *repA*, *repA*, *repB* and 277 bp of *repC*	This work
pMa/AB2	pMPK with 2371 bp *Kpn*I-*Xba*I fragment comprising 269 bp upstream of *repA*, *repA*, *repB* and 1 bp downstream of *repB*	This work
pMa/A2	pMPK with 1543 bp *Kpn*I-*Xba*I fragment comprising 269 bp upstream of *repA*, *repA* and 203 bp of the coding region of *repB*	This work
*repABC* of pRleTA1b		
pMb1-b2	pMPK with 361 bp and 195 bp *Kpn*I-*Xba*I fragments comprising respectively 262 bp and 96 bp upstream of *repA* and 99 bp of *repA*	This work
pMb3	pMPK with 255 bp *Kpn*I-*Xba*I fragment comprising 37 bp upstream of *repA* and 218 bp of *repA*	This work
pMb/ABC2	pMPK with 2677 bp *Kpn*I-*Xba*I fragment comprising 96 bp upstream of *repA*, *repA*, *repB* and 229 bp *repC*, devoid of *parS1* element	This work
pMb/AB1	pMPK with 2472 bp *Kpn*I-*Xba*I fragment comprising 262 bp upstream of *repA*, *repA*, *repB* and 13 bp downstream of *repB*	This work
pMb/AB2	pMPK with 2306 bp *Kpn*I-*Xba*I fragment comprising 96 bp upstream of *repA*, *repA*, *repB* and 13 bp downstream of *repB*, devoid of *parS1* element	This work
pMb/A2	pMPK with 1318 bp *Kpn*I-*Xba*I fragment comprising 96 bp upstream of *repA*, *repA* and 20 bp of *repB*	This work
*repABC* of pRleTA1c		
pMc1-c2	pMPK with 379 bp and 200 bp *Kpn*I-*Xba*I fragment comprising respectively 286 bp and 107 bp upstream of *repA* and 93 bp of *repA*	This work
pMc3	pMPK with 301 bp *Kpn*I-*Xba*I fragment comprising respectively 40 bp upstream of *repA* and 261 bp of *repA*	This work
pMc/ABC2	pMPK with 2733 bp *Kpn*I-*Xba*I fragment comprising 107 bp upstream of *repA*, *repA*, *repB* and 223 bp of *repC*	This work
pMc/AB1	pMPK with 2539 bp *Kpn*I-*Xba*I fragment comprising 286 bp upstream of *repA*, *repA*, *repB* and 49 bp downstream of *repB*	This work
pMc/AB2	pMPK with 2509 bp *Kpn*I-*Xba*I fragment comprising 286 bp upstream of *repA*, *repA*, *repB* and 19 bp downstream of *repB*, devoid of *parS2* element	This work
pMc/AB3	pMPK with 2360 bp *Kpn*I-*Xba*I fragment comprising 107 bp upstream of *repA*, *repA*, *repB* and 49 bp downstream of *repB*, devoid of *parS1* element	This work
pMc/AB4	pMPK with 2330 bp *Kpn*I-*Xba*I fragment comprising 107 bp upstream of *repA*, *repA*, *repB* and 19 bp downstream of *repB*, devoid of both *parS1* and *parS2* elements	This work
pMc/A2	pMPK with 1483 bp *Kpn*I-*Xba*I fragment comprising 107 bp upstream of *repA*, *repA* and 162 bp of *repB*	This work
*repABC* of pRleTA1d		
pMd1-d5	pMPK with 540 bp, 316 bp, 272 bp, 242 bp and 217 bp *Kpn*I-*Xba*I fragments comprising respectively 334 bp, 110 bp, 66 bp, 36 bp and 11 bp upstream of *repA* and 206 bp of *repA*	This work
pMd/ABC2	pMPK with 2832 bp *Kpn*I-*Xba*I fragment comprising 110 bp upstream of *repA*, *repA*, *repB* and 369 bp of *repC*	This work
pMd/AB2	pMPK with 2339 bp *Kpn*I-*Xba*I fragment comprising 110 bp upstream of *repA*, *repA*, *repB* and 30 bp downstream of *repB*	This work
pMd/A2	pMPK with 1488 bp *Kpn*I-*Xba*I fragment comprising 110 bp upstream of *repA*, *repA* and 181 bp of *repB*	This work
pBBR1MCS-5 derivatives		
pBa/AB2	pBBR1MCS-5 with 2371 bp *Kpn*I-*Xba*I fragment of pRleTA1a comprising 269 bp upstream of *repA*, *repA*, *repB* and 1 bp downstream of *repB*	This work
pBa/A2	pBBR1MCS-5 with 1543 bp *Kpn*I-*Xba*I fragment of pRleTA1a comprising 269 bp upstream of *repA*, *repA* and 203 bp of *repB*	This work
pBb/AB1	pBBR1MCS-5 with 2472 bp *Kpn*I-*Xba*I fragment of pRleTA1b containing 262 bp upstream of *repA*, *repA*, *repB* and 13 bp downstream of *repB*	This work
pBb/AB2	pBBR1MCS-5 with 2306 bp *Kpn*I-*Xba*I fragment of pRleTA1b containing 96 bp upstream of *repA*, *repA*, *repB* and 13 bp downstream of *repB*	This work
pBb/A1	pBBR1MCS-5 with 1484 bp *Kpn*I-*Xba*I fragment of pRleTA1b containing 262 bp upstream of *repA*, *repA* and 20 bp of the coding region of *repB*	This work
pBb/A2	pBBR1MCS-5 with 1318 bp *Kpn*I-*Xba*I fragment of pRleTA1b containing 96 bp upstream of *repA*, *repA* and 20 bp of the coding region of *repB*	This work
pBb/B	pBBR1MCS-5 with pQb/B inserted in *Xba*I site	This work
constructs in expression plasmid vectors		
pQ-A/a-d	pQE-30 with 1215 bp *Bam*HI-*Kpn*I and 1203 bp, 1215 bp and 1191 bp *Bam*HI-*Hind*III fragments containing respective pRleTA1a-d *repA* coding sequences without start codon	This work
pQa-b/B, pQd/B	pQE-30 with 1026 bp, 993 bp and 999 bp *Bam*HI-*Hind*III fragments containing respective *repB* coding sequence of pRleTA1a-b and pRleTA1d without start codon	This work
pETc/B	pET-32a(+) with 984 bp *Bam*HI-*Hind*III fragment containing *repB* coding sequence of pRleTA1c without start codon	This work
BACTH system plasmids and constructs carrying *repA* and *repB* of pRleTA1b		
pUT18	Two-hybrid plasmid for *cyaA*T18 fusion construction, Amp^r^	[42]
pUT18C	Two-hybrid plasmid for *cyaA*T18 fusion construction, Amp^r^	[42]
pKT25	Two-hybrid plasmid for *cyaA*T25 fusion construction, Km^r^	[42]
pKNT25	Two-hybrid plasmid for *cyaA*T25 fusion construction, Km^r^	[42]
pUT18C-zip	Two-hybrid control plasmid	[42]
pKT25-zip	Two-hybrid control plasmid	[42]
RepA/RepB-T18	pUT18 carrying 1200 bp and 990 bp *Xba*I-*Kpn*I fragments of *repA/repB* coding sequences devoid of start and stop codons	This work
T18-RepA/RepB	pUT18C carrying 1203 bp and 994 bp *Xba*I-*Kpn*I fragments of *repA/repB* coding sequences devoid of start and stop codons	This work
T25-RepA/RepB	pKT25 carrying 1204 bp and 994 bp *Xba*I-*Kpn*I fragments of *repA/repB* coding sequences devoid of start and stop codons	This work
RepA/RepB-T25	pKNT25 carrying 1203 bp and 990 bp *Xba*I-*Kpn*I fragments of *repA/repB* coding sequences devoid of start and stop codons	This work
T25-RepA_79-401_	pKT25 carrying 993 bp *Xba*I-*Kpn*I fragment of truncated *repA*, encoding the 79–401 amino acid residues of RepA	This work

Abbreviations: Str^r^, streptomycin resistance; Rif^r^, rifampicin resistance; Km^r^, kanamycin resistance; Gm^r^, gentamicin resistance; Amp^r^, ampicillin resistance; Tet^r^, tetracycline resistance, Cm^r^, chloramphenicol resistance.

### DNA manipulation techniques

Standard techniques were employed for genomic DNA and plasmid isolation, PCR, molecular cloning, agarose gel electrophoresis and transformation [32]. Restriction and ligation reactions were conducted according to conditions specified by the manufacturer of the enzymes (Thermo Scientific). PCR was performed using high fidelity DNA polymerase (Sigma-Aldrich). The primers used in this work were obtained from Genomed (Warsaw, Poland) and are listed in S1 Table. Automatic sequencing was performed using BigDye Terminator Cycle Sequencing Kit and ABI PRISM 310 or Applied Biosystems 3500 Genetic Analyzers (Applied Biosystems).

### Construction of *rep*-*lacZ* transcriptional and translational fusions

Transcriptional activity of *in silico* predicted promoters (P*rep*) of *repABC* cassettes of four RtTA1 plasmids was studied in a series of transcriptional fusions with promoterless *lacZ* gene in the pMPK reporter vector, which is pMP220 derivative [34] constructed in this work (Table 1). To construct these plasmids appropriate DNA fragments were PCR amplified using an upstream primer for the putative promoter region equipped with *Kpn*I restriction site and the reverse primer, annealing within the coding sequence of relevant *repA* gene equipped with *Xba*I site, and cloned into pMPK. The recombinant plasmids were transferred into *A*. *tumefaciens* GMI9023 (*Atu*) by electrotransformation and the promoter activities were determined by measurement of the β-galactosidase activity. To define if *repABC* genes form an operon, the PCR amplified DNA fragments comprising P*rep*, *repA*, *repB* and 5' end of *repC* were transcriptionally fused with *lacZ* in *Kpn*I-*Xba*I sites of pMPK, and reporter activity was measured in *Atu* to avoid presumable incompatibility of such constructs in RtTA1. To reveal potential autoregulation of *repABC* operons and involvement of centromere-like *parS* sequences in this process, a set of deletion derivatives of particular *repABC* operons transcriptionally fused with *lacZ* in pMPK were constructed. The constructs comprised either P*rep*, *repA* and *repB* (with or without respective *parS* elements) or only P*rep* and *repA*. The respective DNA fragments were PCR amplified, cloned into *Kpn*I-*Xba*I sites of pMPK and β-galactosidase activity was assayed in *Atu*. To test the potential ability of RepA and RepB to exert negative autoregulation on the operon when provided *in trans*, the *repA* and *repAB* genes of plasmids pRleTA1b and pRleTA1a were cloned into *Kpn*I-*Xba*I sites of pBBR1MCS-5 [35]. To reveal the contribution of RepB alone on the *repABC*/b expression, *Bam*HI-*Hind*III fragment containing *repB* coding sequence of pRleTA1b was cloned into pQE-30, and the recombinant plasmid was subsequently digested with *Xba*I and recloned into pBBR1MCS-5, resulting in the pBb/B. All constructs were verified by sequencing.

### β-Galactosidase activity measurements

The *A*. *tumefaciens* strains carrying the *lacZ* transcriptional fusions in pMPK were grown overnight in 79CA medium in the presence of kanamycin. The cultures were diluted in fresh M1 medium supplemented with a vitamin mixture according to Brown and Dilworth [36] and grown to mid-log phase. The *E*. *coli* strains were grown and diluted in LB medium. The level of *lacZ* expression was determined in Miller units, by assaying β-galactosidase activity with the ONPG (2-Nitrophenyl-β-D-galactopyranoside, MP Biomedicals) as a substrate, as described by Miller [37].

### Overproduction and purification of recombinant RepA proteins

For the expression and purification of recombinant RepA/a-d proteins with N-terminal His_6_-tag the relevant *repA* genes were PCR amplified using appropriate primer pairs (S1 Table), high fidelity DNA polymerase and RtTA1 genomic DNA as a template. Amplification products were cloned into *Bam*HI and *Hind*III sites or *Bam*HI and *Kpn*I sites of pQE-30 vector (Qiagen) resulting in pQ-A/b-d and pQ-A/a recombinant plasmids, respectively. The following *E*. *coli* strains were used for propagation of the expression constructs: M15[pREP4] for pQ-A/a, pQ-A/b and pQ-A/d plasmids or JM101 for pQ-A/c. Strains carrying recombinant plasmids expressing RepA were grown at 37°C in LB broth containing appropriate antibiotics. IPTG (isopropyl-β-D-thiogalactopyranoside, Thermo Scientific) was then added to a final concentration of 0.1 mM when the cultures reached an OD_600_ of 0.6 (pQ-A/a, pQ-A/d) or 1.0 (pQ-A/b, pQ-A/c), and incubation was continued for 5 hours or overnight, respectively. The cells were then chilled on ice, harvested by centrifugation, resuspended in lysis buffer (50 mM NaH_2_PO_4_, 300 mM NaCl, pH 8.0) supplemented with lysozyme to 1 mg/ml (MP Biomedicals) and protease inhibitor cocktail (Sigma-Aldrich), and incubated on ice for 30 min. The suspensions containing His_6_-RepA/a were additionally treated with DNase I (5 μg/ml, MP Biomedicals) on ice for 10 min. The cells were then disrupted using the FRENCH Pressure Cell Press (Thermo Scientific) and centrifuged at 10,000 × *g* for 20 min at 4°C to separate the soluble and cellular debris fractions. The supernatants were then subjected to His_6_-tagged protein purification via Co^2+^ affinity chromatography using TALON Metal Affinity Resin (Clontech Laboratories, Inc.). The samples were mixed with affinity resin, gently agitated on ice in order to allow the His_6_-tagged proteins to bind the resin, then washed twice with wash buffer I (50 mM phosphate, 300 mM NaCl, 10 mM imidazole, pH 8.0) and then once with wash buffer II (50 mM phosphate, 300 mM NaCl, 20 mM imidazole, pH 8.0). In the case of His_6_-RepA/a, extra-washing step with wash buffer III (50 mM phosphate, 1 M NaCl, 10 mM imidazole, pH 8.0) just before rinsing with wash buffer II was necessary, in order to get rid of DNA bound to proteins. Next, elution buffer (50 mM phosphate, 300 mM NaCl, 250 mM imidazole, pH 8.0) was applied and the fractions containing His_6_-RepA proteins were pooled and stored in 4°C. Samples were analyzed by SDS-PAGE, Western immunoblotting with anti-His_6_ antibodies (Roche) and quantitated using Qubit Fluorometer (Invitrogen).

### Overproduction and purification of recombinant RepB proteins

The relevant *repB* genes were PCR amplified and cloned into *Bam*HI and *Hind*III sites of pQE-30 vector (Qiagen) (pQa/B, pQb/B, and pQd/B plasmids) or pET-32a(+) (Novagen) (pETc/B plasmid). *E*. *coli* M15[pREP4] and Rosetta 2(DE3)pLysS strains were used for propagation of the pQE-30-based and pET-32a(+)-based expression constructs, respectively. Strains carrying recombinant plasmids expressing RepB were grown at 37°C in LB broth containing appropriate antibiotics. IPTG was then added to a final concentration of 0.5 mM when the cultures reached an OD_600_ of 0.6 and incubation was continued for 5 hours. Further steps in isolation and purification of RepB recombinant proteins were performed as described above for His_6-_RepA proteins.

### Electrophoretic mobility shift assay (EMSA)

Non-radioactive electrophoretic mobility shift assay was used for analysis of recombinant RepA proteins binding with DNA. The target DNA fragments subjected to EMSA were PCR amplified with appropriate primers (S1 Table), high fidelity DNA polymerase and RtTA1 genomic DNA as a template. The target fragments encompassed the promoter and operator regions of the individual RtTA1 *repABC* cassettes, as well as their 5’ and 3’ sequential deletions of various length. The 148 bp non-specific control PCR product comprising fragment of the Km^r^ gene of the pBBR1MCS-2 [35] was employed in these experiments. DNA binding reaction was performed at 28°C for 30 min by incubating 5–25 ng of target DNA and 5–25 ng control DNA with 10–100 pmol of purified recombinant RepA proteins in binding buffer (10 mM Tris-HCl [pH 8.0], 50 mM NaCl, 1 mM EDTA, 5 mM MgCl_2_, 5 mM DTT, 100 μg bovine serum albumin and 5% [v/v] glycerol). The final volume of the reaction mixture was 20 μl. The ATP or ADP was present in the binding reaction when necessary in final concentration of 0.1, 1 or 2 mM. After incubation, 1 μl of loading dye (0.03% [w/v] xylene cyanol, 0,03% [w/v] bromophenol blue, 60% [v/v] glycerol) was added, and the samples were immediately loaded and separated by electrophoresis at room temperature on native 10% polyacrylamide gel (79:1) in TB buffer supplemented with 1 mM MgCl_2_ The gels were then stained with SYBR Green (Sigma-Aldrich) or ethidium bromide (Sigma-Aldrich) and exposed to UV.

### Cross-linking experiments

The His_6_-RepA proteins (0.1–0.2 nM) stored in elution buffer were subjected to cross-linking experiments. DMP (dimethyl pimelimidate, Sigma-Aldrich) was added to the reactions at final concentrations of 0.5 mM, 1 mM, 10 mM and 25 mM. The final reactions volume was between 15 and 25 μl. The cross-linking reactions were incubated for 1 h at 28°C and then stopped by the addition of 1 μl of 0.5 M Tris-HCl (pH 6.8) followed by addition of 5× SDS loading buffer. The samples were heated at 95°C for 5 min and analyzed by SDS-PAGE followed by Western analysis with anti-His_6_ antibodies (Roche). In the time course experiment the 10 mM DMP concentration was fixed and the protein samples were incubated at 28°C from 30 s to 120 min. 2 mM ATP or ADP and 10–50 ng target Op DNA fragment or non-specific DNA competitor was added when needed.

### Bacterial Two-Hybrid (BACTH) complementation assays

To construct the recombinant plasmids used in the BACTH complementation assays, the genes coding for the RepA/b, RepB/b proteins or RepA/b subdomain were PCR amplified, using appropriate primers listed in S1 Table and the RtTA1 genomic DNA as a template. Amplified DNA fragments were digested with appropriate restriction enzymes and subcloned into the corresponding sites of the 'bait'/'prey' vectors: pUT18, pUT18C, pKT25 and pKNT25, in DH5α strain. The resulting recombinant plasmids expressed hybrid proteins in which the polypeptides of interest were fused to the N-terminus of T18 and T25 fragments of adenylate cyclase in pUT18 and pKNT25 or to the C-terminus of T25 and T18 fragments in pKT25 and pUT18C, respectively. DNA sequences of the cloned genes in all recombinant plasmids were verified by sequencing. Next, *E*. *coli* DHM1, which carries a 200-bp deletion within the *cya* gene, was sequentially transformed with all the combinations of recombinant BACTH plasmids. Initial scoring of potential interactions was achieved on the LB plates containing ampicillin, kanamycin, X-Gal (40 μg/ml) and IPTG (0.5 mM), which were incubated at 30°C for 48 h. For a quantitative measurement, bacteria were grown in LB broth in the presence of 0.5 mM IPTG and appropriate antibiotics at 30°C for 14 to 16 h. Measurement of β-galactosidase activity was performed according to a procedure described by Miller [37]. pKT25-zip and pUT18C-zip derivatives of 'bait'/'prey' vectors, carrying gene fragments encoding GCN4 leucine zipper motifs, and empty 'bait'/'prey' vectors pKT25, pKNT25, pUT18C and pUT18 were used as the positive and negative control, respectively. For reproducible and robust results, only freshly transformed colonies were used.

### Bioinformatics tools

Sequence data were analyzed with Lasergene analysis software (DNASTAR, Inc.). The promoter regions were analyzed using the DBGP server at University of California, Berkeley [38] and Promoter 2.0 algorithm [39]. For protein secondary structure analyses the PSIPRED prediction method was used [40]. Putative helix-turn-helix (HTH) motifs were predicted using GYM 2.0 [41].

## Results

### *repABC* genes of the individual plasmids of RtTA1 form operons negatively regulated by RepA and RepB

**Fig 1:**
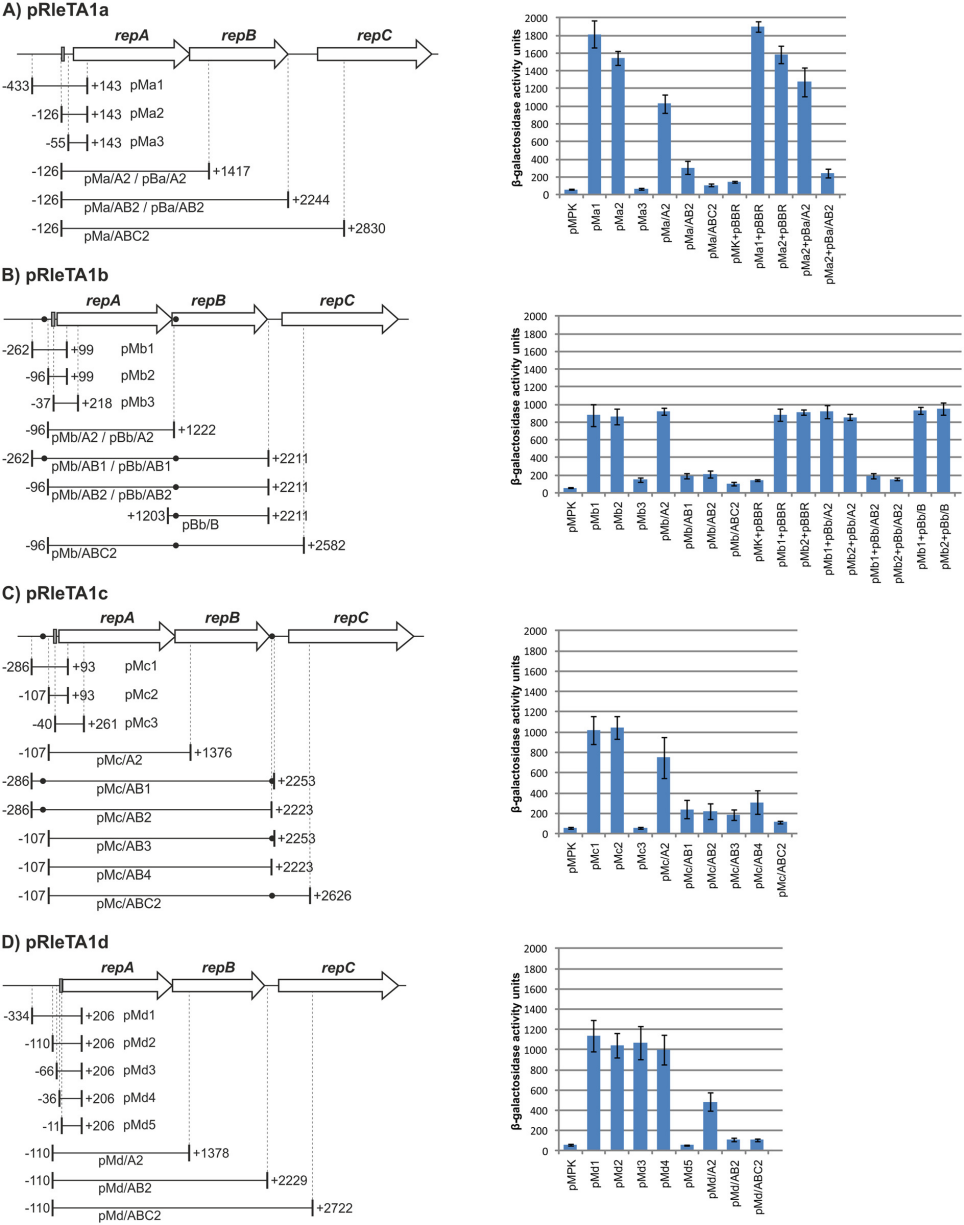
Transcriptional activity of *repABC* operons of *Rhizobium leguminosarum* bv. *trifolii* TA1 plasmids: (A) pRleTA1a, (B) pRleTA1b, (C) pRleTA1c, and (D) pRleTA1d. Left panels show schematic genetic organization of the *repABC* cassettes. White arrows represent *repA*, *repB* and *repC* genes, and white broken arrows depict *repA* genes. Black dots show the position of *parS*-sites. The identified promoters of *repABC* operons were marked as grey rectangular boxes. The respective DNA fragments necessary for particular promoter identification, operon structure assignment, as well as their sequential deletions used for operon regulation studies, which were cloned into pMPK reporter and pBBR1MCS-5 vectors, were shown as black lines with positions indicated relative to *repA* start codon. The following system was applied for pMPK- and pBBR-1MCS5-based constructs nomenclature: e.g. in pMa/A2 recombinant plasmid the first two letters (pM) mean the shortcut of vector name (pMPK in this case), the next small letter 'a' means that it is derivative of pRleTA1a *repABC* cassette, the capital letter A followed by slash means that the cloned fragment comprise entire *repA* gene (in the case of pMa-d/ABC2 constructs the cloned fragments encompass entire *repA* and *repB* genes and fragment of *repC* fused with *lacZ*). Respectively, pBa/A2 means the same fragment recloned to the pBBR-1MCS5. Right panels represent β-galactosidase activities of respective *lacZ* transcriptional fusions measured in *A*. *tumefaciens* and expressed in Miller units. Each value (with standard deviation—extended bars) is the average of at least three independent measurements.

*In silico* promoter prediction suggested operon organization of all RtTA1 *repABC* regions, with the putative promoter located upstream of the *repA* genes [31]. Moreover, in the putative promoter regions of all RtTA1 *rep* cassettes, operator-like palindromes were identified suggesting complex regulation of *repABC* expression. To study the transcription of the *repABC* genes of individual RtTA1 plasmids and regulation of the putative *repABC* operons with special attention to the contribution of RepA in this process, series of *rep-lacZ* transcriptional fusions were constructed and analysed (Fig 1).

#### Mapping of the promoters of *repABC* cassettes

First, promoters of individual RtTA1 *repABC* cassettes were mapped. DNA fragments comprising regions upstream of particular *repA* genes sequentially shortened at the 5’-end were PCR amplified and cloned into pMPK, creating a set of *rep*-*lacZ* transcriptional fusions (Fig 1A–1D), which were introduced into *Atu*. The reporter activities differed in fusions comprising promoters of particular *repABC* cassettes. The highest relative level of β-galactosidase was estimated for the *repABC* promoter of pRleTA1a (Fig 1A), while the LacZ activities of other *rep* regions were almost twofold lower (Fig 1A–1D). The LacZ activity of the pMa1-a2 was high, while in the case of the pMa3 it was drastically reduced. These results allowed mapping of the *repA* upstream elements essential for transcription initiation of the pRleTA1a *rep* cassette, which was located between -126-55 bp (Fig 1A). Noteworthy, pMa2 was able to express the reporter gene in *E*. *coli*, indicating that the RtTA1 *rep* promoters can be recognized by the *E*. *coli* transcription machinery. A sequence [TTGCCA(N_16_)TAAGTT] with some similarity to the *E*. *coli* σ^70^ promoter with a putative -35 and -10 consensus box was recognized in the fragment cloned into pMa2.

The promoters of other plasmid *rep* cassettes were mapped by a similar approach. The reporter activity of pMb1-b2 and pMc1-c2 was high (Fig 1B and 1C), but was drastically reduced in the case of the pMb3 and pMc3, allowing mapping of the *repA* upstream elements essential for transcription initiation of relevant *rep* cassettes, which were located between -96-37 bp and downstream of -107 bp for pRleTA1b and pRleTA1c plasmids, respectively. Similarly to pMa2, pMb2 and pMc2 were able to express the reporter gene in *E*. *coli*, and again sequences with some similarity to the *E*. *coli* σ^70^ promoter with putative -35 and -10 consensus boxes were recognized in the fragments cloned into pMb2 [GTGTCA(N_22_)TATGTT] and pMc2 [TCGGCA(N_18_)CAAAAT].

Surprisingly, to map the region essential for transcription initiation of the pRleTA1d *rep* cassette, five *lacZ* transcriptional fusions were required. The β-galactosidase activity of four constructed plasmids denoted as pMd1-d4 comprising a sequentially shortened 5’-end of the hypothetical promoter was still high up to pMd5, in which the LacZ activity was substantially reduced (Fig 1D). In the *repA* upstream region of pRleTA1d, several strong promoters were predicted. pMd1-d4 were able to express the reporter gene in *E*. *coli*, and within the cloned fragments, sequences similar to the *E*. *coli* σ^70^ promoter consensus was recognized: in the pMd1 [CTGACG(N_17_)TAAATT] and pMd2 [TTGTAA(N_20_)TATAGT], and in the pMd3 and pMd4 [TTCAAA(N_17_)GAAAAA]. It was concluded that the *repABC*/d promoter was most probably located downstream of the -36 bp position.

#### Operon organization of *repABC* cassettes

The operon organization of the RtTA1 *repABC* cassettes was verified subsequently with another set of pMPK-based *lacZ* transcriptional fusions (Fig 1A–1D). The pMa-d/ABC2 plasmids with fragments comprising the promoters defined above, *repA*, *repB*, ctRNA gene, and part of *repC*, to which promoterless *lacZ* was fused, were still able to express β-galactosidase. The level of LacZ activity was very low, however, it differed significantly (p<0.001) from the control pMPK, indicating that the *repABC* cassettes constitute operons (Fig 1A–1D). Moreover, the low reporter activity observed in all the tested *repABC-lacZ* fusions strongly suggested that RepA and/or RepB participated in the autorepression of the operons.

#### The role of RepA and RepB in operon autorepression

To examine the role of RepA and RepB in autorepression, a series of plasmids, which contained the respective promoters and expressed either RepA alone or both RepA and RepB proteins were tested for the ability to repress the transcription of respective operons. In the pMPK-based constructs containing the *repA* gene and expressing RepA *in cis* from its own promoter (pMa-d/A2 series), the β-galactosidase activity was decreased by approximately 30% for the *repABC*/a cassette, 20% for *repABC*/c, and 50% for *repABC*/d. However, it was not affected in the case of *repABC*/b, compared to the LacZ activity of the pMa-c2 and pMd-4 plasmids (Fig 1A–1D). This diverse RepA effect on the transcriptional activity of the particular *rep* operons once again demonstrated some dissimilarity between the individual *repABC* cassettes. In turn, in the pMPK derivatives with cloned *repAB* genes (without the ctRNA gene located downstream of the respective *repB*) and expressing RepA and RepB *in cis* from the promoter preceding *repA* (pMa-d/AB series), the LacZ activity was significantly diminished in the case of all the *repAB-lacZ* fusions (Fig 1A–1D). We have concluded that RepA and RepB acting together repress the individual *repABC* operons, resulting in negative autoregulation. We have also tested the ability of the RepA and RepB proteins to exert negative autoregulation of the operon when provided *in trans*. The *repA* and *repAB* genes of two plasmids pRleTA1a and pRleTA1b were cloned into pBBR1MCS-5 (Fig 1A and 1B). Introduction of pBa/A2 or pBb/A2 plasmids that expressed RepA into *Atu* harbouring pMa2 or pMb2, respectively, moderately repressed the *lacZ* expression of the pMa2 fusion (the level of the reporter activity was comparable to that observed in pMa/A2) and had almost no effect on the reporter activity in pMb2 (as in the case of pMb/A2) (Fig 1A and 1B). However, the activity of β-galactosidase in the relevant P*rep*-*lacZ* fusions was substantially decreased when a second vector, expressing both RepA and RepB (pBa/AB2 or pBb/AB1 and pBb/AB2), was introduced into *Atu* with pMa2 or pMb2, respectively (Fig 1A and 1B). Concomitantly, when pBb/B (Fig 1B) expressing only RepB/b was introduced into *Atu* with pMb1 or pMb2, the activity of β-galactosidase of neither of these two *lacZ* fusions was affected. These results show that RepA is important for autorepression but strong repression of the individual RtTA1 *repABC* operons is possible only in the presence of both RepA and RepB proteins, which can be provided *in trans*, but not in the presence of RepB only.

#### The role of *parS* elements in *repABC* operon expression

To study the role of *parS* centromere-like elements in the RtTA1 *repABC* operon expression, the pMPK-based transcriptional fusions with fragments containing *repAB* genes of the pRleTA1b and pRleTA1c but deleted for respective *parS* sequences were examined (Fig 1B and 1C). In each of these *repABC* cassettes, two *parS* sites were previously found, which introduced *in trans* into the RtTA1 genome exerted incompatibility against respective parental plasmids [31]. In the *lacZ* fusions comprising promoters, *repA*, *repB*, and just one *parS* site irrespective of its location, i.e. within the *repB* coding sequence (pMb/AB2), upstream of *repA* (pMc/AB2), or downstream of *repB* (pMc/AB3), the β-galactosidase activity was low (Fig 1B and 1C). The LacZ activity measured in *Atu* cells bearing the pMb/AB1 or pMc/AB1 constructs with two *parS* elements was also low and comparable to the β-galactosidase activity of the construct with just one centromere-like element (pMb/AB2 and pMc/AB2 or pMc/AB3) (Fig 1B and 1C). On the other hand, *lacZ* expression in the pMc/AB4 deleted for both *parS* sites was only slightly elevated in relation to pMc/AB1, pMc/AB2, or pMc/AB3 (Fig 1C). It was concluded that presence of the *parS* element was not mandatory to achieve strong repression of the *repABC* operon, whereas RepA-RepB protein interaction is crucial for the regulation.

Summarizing, these results showed that the *repABC* genes of all the RtTA1 plasmids were organized as operons and were negatively regulated by cooperating RepA and RepB proteins. Despite the similarity in the genetic organization of the individual RtTA1 *rep* operons, they differed slightly with respect to the location of the individual promoter, their transcriptional activity that was twofold higher for pRleTA1a in comparison to other plasmids, and response to the RepA regulatory protein.

### Individual RtTA1 RepA proteins specifically bind to the operator for autorepression of operon

**Fig 2:**
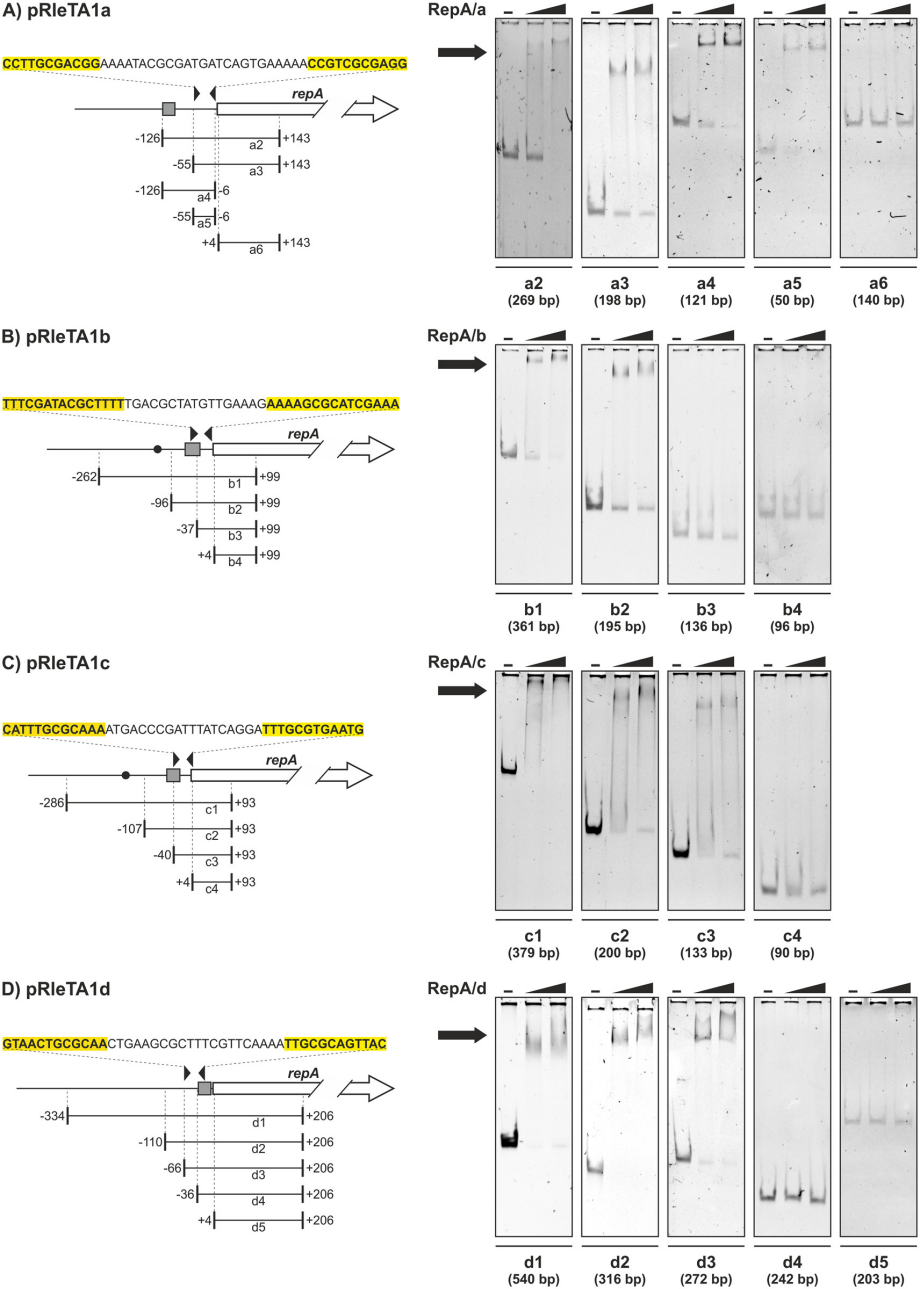
Mapping of operator sequences of *repABC* operons of RtTA1 plasmids: (A) pRleTA1a, (B) pRleTA1b, (C) pRleTA1c, and (D) pRleTA1d. Left panels show schematic depictions of the region upstream of *repA* of *repABC* operons. The respective promoters (grey rectangle), operators—imperfect palindromes (black inverted triangles), *parS* elements (black dots) and *repA* genes (white broken arrow) were shown. The sequence of each palindrome is highlighted in yellow. Length and relative position of DNA fragments used in operator (Op) mapping were shown with respect to *repA* start codon. Right panels represent EMSA results with recombinant His_6_-RepA and respective DNA fragments (5–25 ng). Black triangles indicate the increasing concentration of particular His_6_-RepA protein (10 and 100 pmol) present in DNA binding reaction. Black arrows indicate the position of the retarded DNA bands.

As demonstrated above, individual RepA can, at least partially, repress its own transcription. In each of the four RtTA1 plasmids, a putative operator sequence (Op) was found upstream of the *repA* gene initial codon. The operons contained imperfect palindromes, which in the case of the *repABC*/b, *repABC*/c, and *repABC*/d cassettes partially overlapped the identified promoters (Fig 2A–2D).

**Fig 3:**
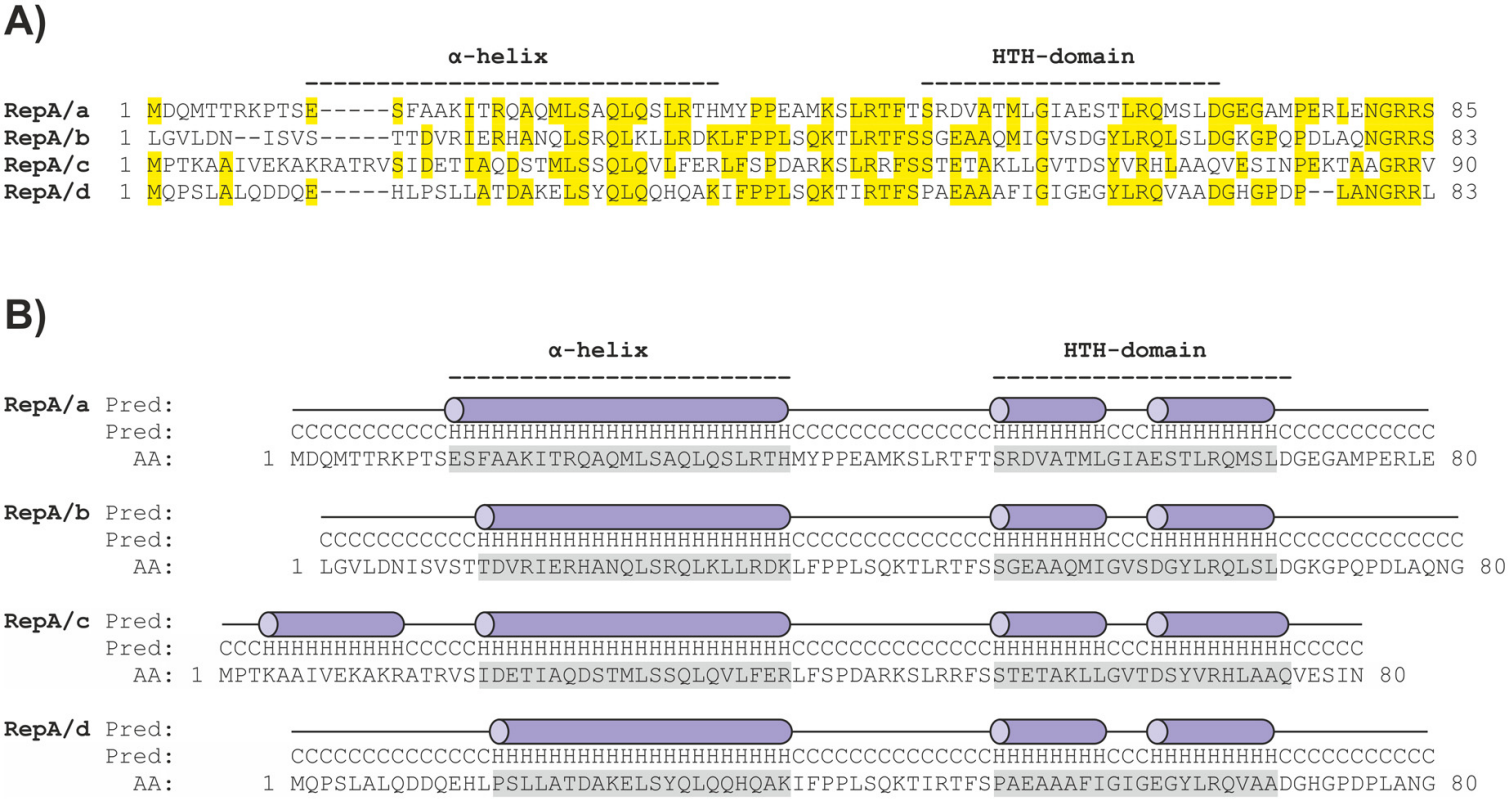
Amino acid sequence analysis of RepA proteins of RtTA1 plasmids. (A) Multiple alignment of N-terminal sequences of RepA/a-d. Identical amino acid residues were marked in yellow. (B) Secondary structure predictions of N-termini of individual RepA proteins. The regions constituting the α-helices responsible for RepA dimerization and helix-turn-helix (HTH) DNA binding domains were presented as blue cylinders and highlighted in grey.

**Fig 4:**
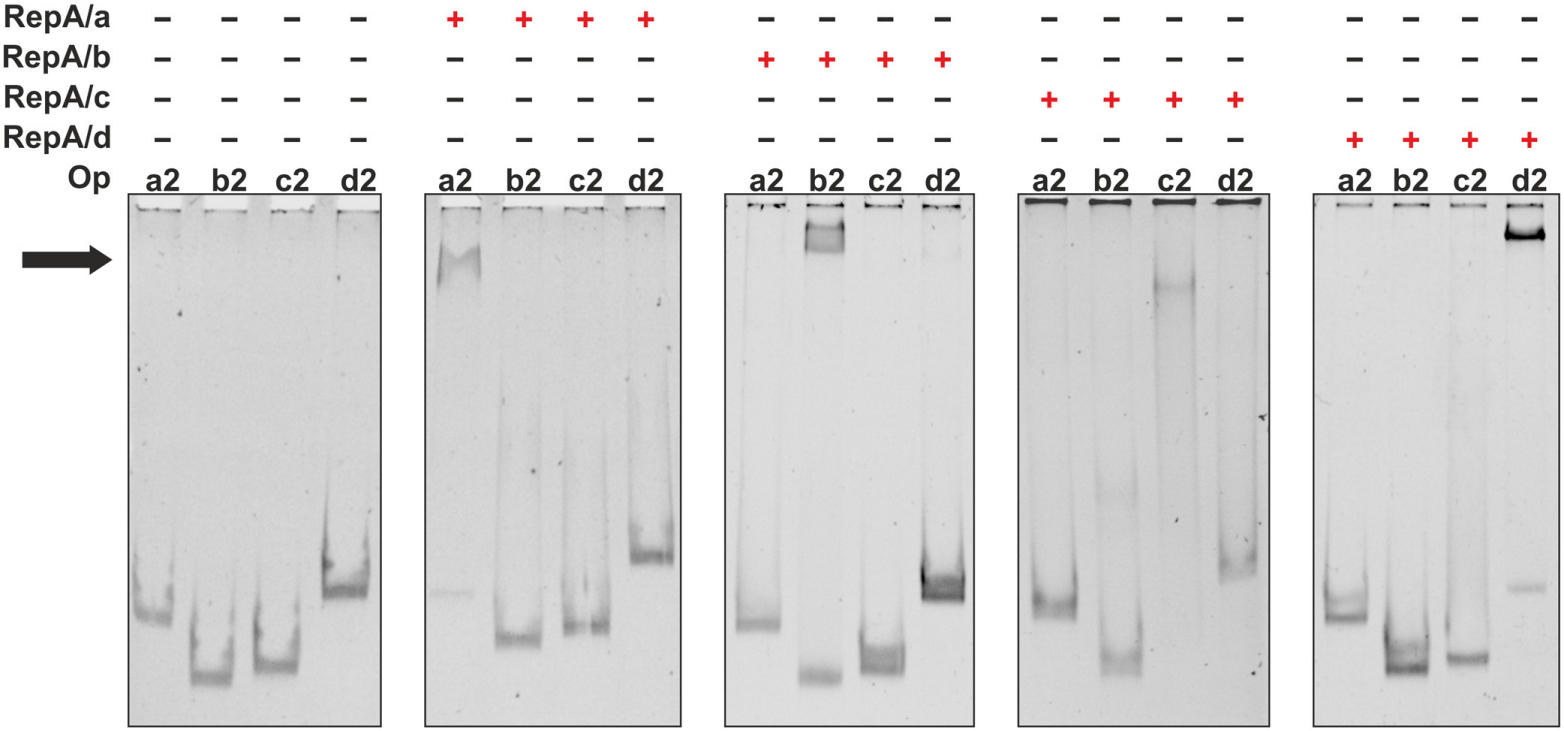
EMSA based analysis of His_6_-RepA proteins binding specificity to operator (Op) sequences originating from parental and non-parental plasmids. DNA fragments (15 ng) (identical as those marked in Fig 2) designated a2, b2, c2 and d2 comprised mapped operators. Red '+' means presence of respective His_6_-RepA protein (100 pmol) in the binding reaction. Black arrow indicates position of the retarded DNA bands.

In the N-terminal part of the individual RepA proteins, elongated α-helices and helix-turn-helix (HTH) DNA binding motifs were predicted (Fig 3). To study the binding of RepA proteins to putative Op DNA sequences, we undertook EMSA analyses. Particular RepA were overexpressed in *E*. *coli* as N-terminally His_6_-tagged recombinant proteins, purified by affinity chromatography under native conditions, and used in series of non-radioactive EMSA with PCR-amplified DNA fragments of various lengths, comprising putative Op sequences of individual *repABC* operons (Fig 2A–2D). Using this *in vitro* approach for all of the His_6_-RepA RtTA1 proteins, their ability for binding to their own Op element was demonstrated (Fig 2A–2D). Moreover, the length of the operator sequence necessary for respective RepA binding was mapped and ranged from -55 to -6 bp for *repABC*/a, downstream of -96 bp for *repABC*/b, downstream of -40 bp for repABC/c, and downstream of -66 for *repABC*/d relative to the *repA* ATG codon (Fig 2A–2D). Noteworthy, no cross reactivity between individual RepA proteins and Op elements from non-parental *repABC* cassettes was demonstrated (Fig 4). The RepA proteins bound to their own *repABC* operator sites in a very specific manner, which is likely to correlate with operon autorepression. The negative regulation of transcription of RtTA1 *rep* operons was strongly enhanced by RepB, as demonstrated above. We performed the EMSA with respective recombinant RepB proteins and DNA fragments denoted a5, b2, c3, and d3 (Fig 2), comprising the minimal-length DNA segments upstream of *repA* necessary for respective His_6_-RepA binding, but no shifted bands were observed in this assay. These results indicate that individual RepB proteins may exert their corepressor action by binding to corresponding RepA (described later) rather than directly to the Op region.

**Fig 5:**
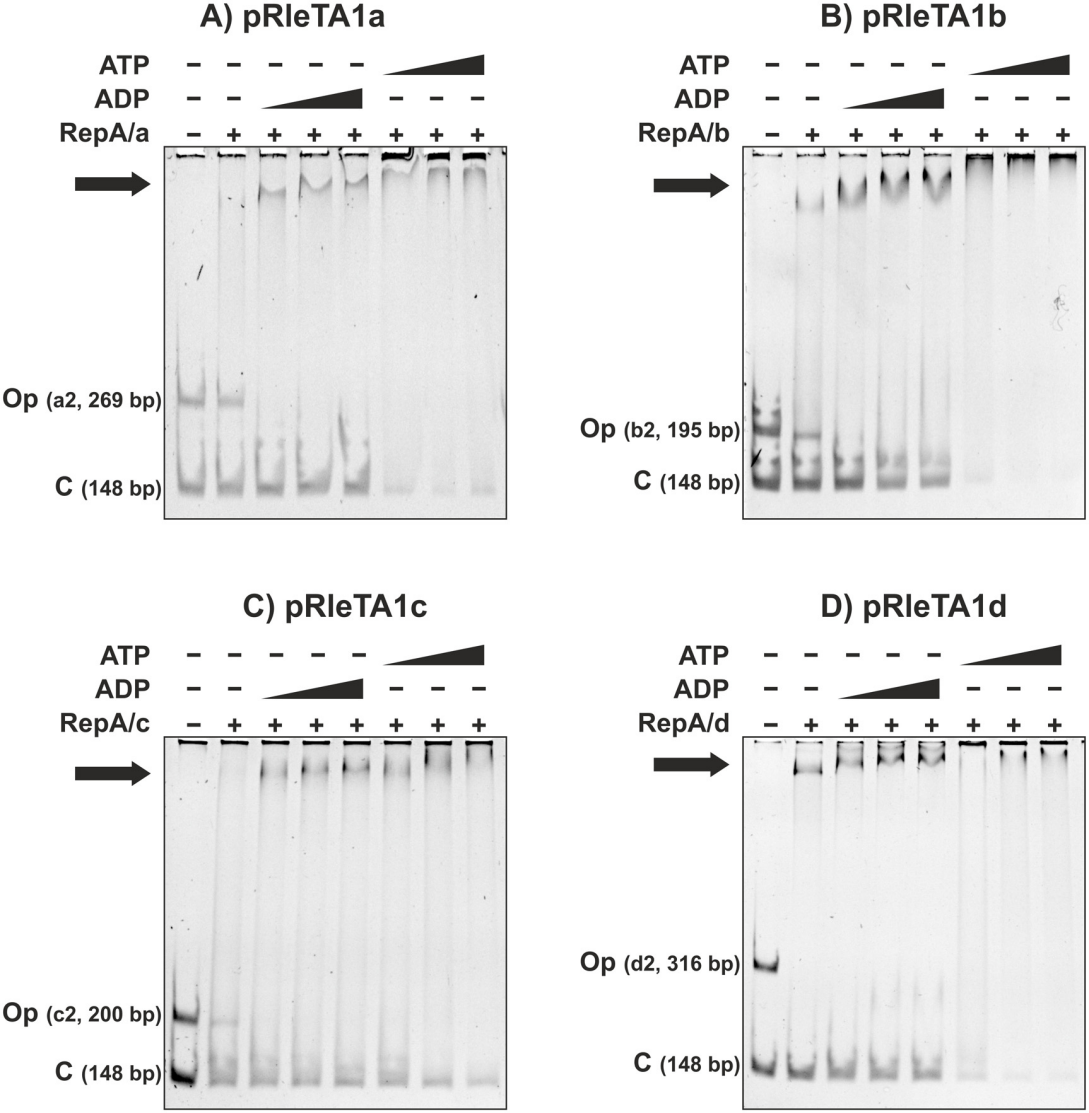
Analysis of ADP/ATP role in the DNA binding specificity of RepA proteins: (A) pRleTA1a, (B) pRleTA1b, (C) pRleTA1c, and (D) pRleTA1d. 'Op' indicates DNA fragment (15 ng) comprising mapped operator sequences of individual *repABC* operons, while 'C' means non-specific DNA competitor (15 ng) (148 bp DNA fragment of the gene coding for Km^r^ of the pBBR1MCS-2). The Op fragments (a2, b2, c2, d2) correspond to the ones shown in Fig 2. Black triangles indicate the increasing concentration of ADT/ATP (0.1, 1, and 2 mM) while '+' means presence of respective His_6_-RepA (10 pmol) protein in the binding reaction. Black arrows indicate the position of the retarded DNA bands.

The role of ATP/ADP in the DNA binding specificity of RepA proteins was examined in the EMSA, in which two DNA fragments were used: a specific—including the respective Op sequence—and a non-specific competitor (an internal fragment of the Km^r^ gene of the pBBR1MCS-2) (Fig 5). For each of the recombinant RtTA1 His_6_-RepA proteins, the addition of ATP stimulated its non-specific DNA binding, while in the presence of ADP the individual RepA bound specifically to Op sequences (Fig 5A–5D). These results show that, in the presence of ADP, individual RepA bind specifically to their own Op sequence for *rep* operon repression.

### RepA proteins are able to self-associate *in vitro* in an ATP/ADP-dependent manner and differ in the oligomerization pattern

**Fig 6:**
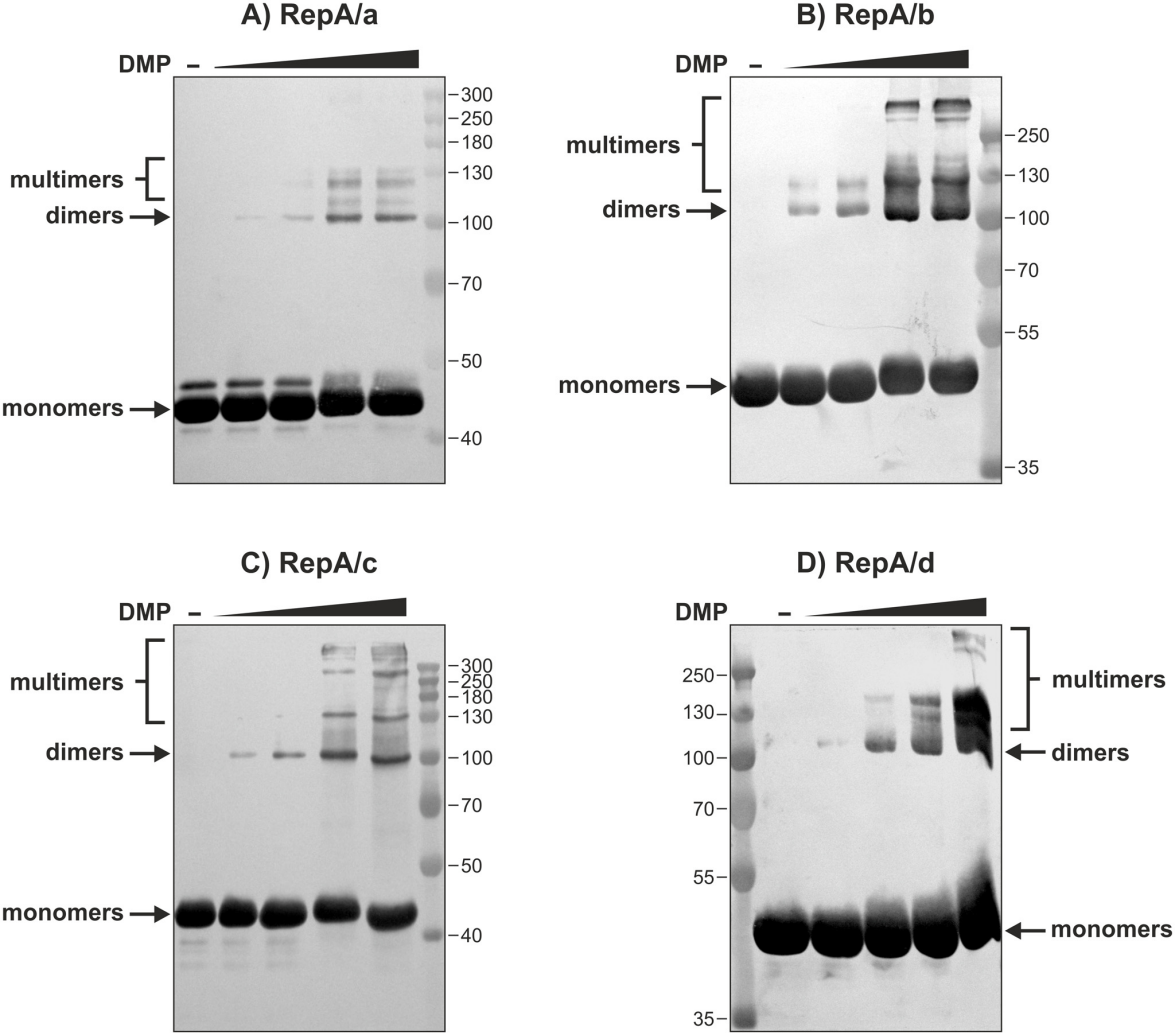
Cross-linking analysis of His_6_-RepA/a-d. His_6_-tagged RepA proteins (0.1–0.2 nM) were incubated with increasing concentration of DMP (0.5 mM, 1 mM, 10 mM, and 25 mM), separated on 10% SDS-PAGE and visualized by Western blot with anti-His_6_ antibodies. The thin lines indicate the marker bands whose molecular masses are expressed in kDa. Arrows indicate different species formed by RepA proteins corresponding to monomers and dimers, while the position of multimers was marked with brackets.

Cross-linking experiments with dimethyl pimelimidate (DMP) were performed to characterize the ability of RtTA1 RepA to oligomerize. When individual His_6_-RepA/a-d were treated with a solution with an increasing concentration of DMP, cross-linked dimeric species were unambiguously detected (Fig 6A–6D). At a higher DMP concentration, bands corresponding to multimeric fractions were observed for all of the RepA proteins (Fig 6).

**Fig 7:**
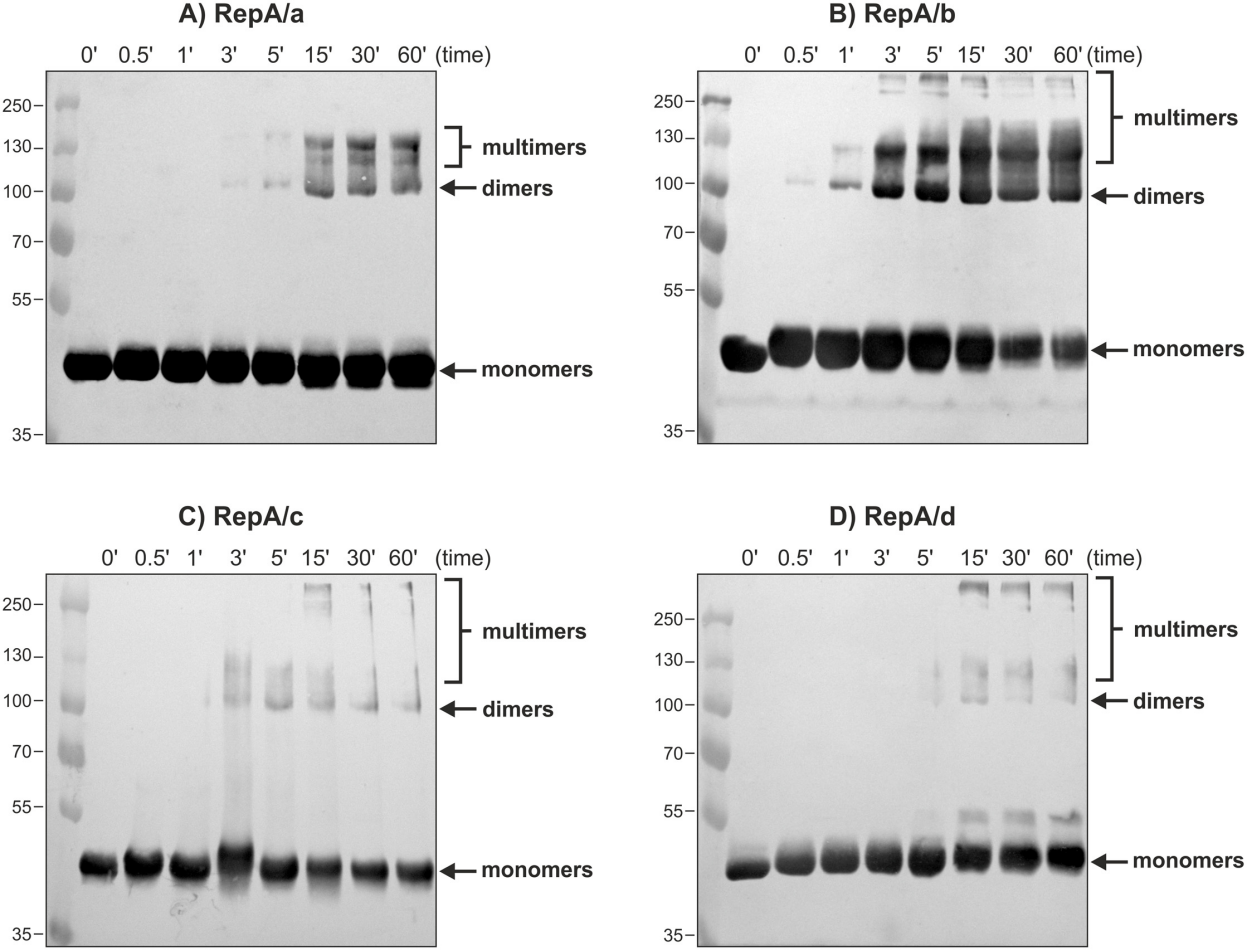
Time course cross-linking analysis of His_6_-RepA/a-d. His_6_-tagged RepA proteins (0.1–0.2 nM) were incubated with DMP concentration fixed at 10 mM, at 28°C, from 30 s to 120 min, separated on 10% SDS-PAGE and visualized by Western blot with anti-His_6_ antibodies. The thin lines indicate the marker bands whose molecular masses are expressed in kDa. Arrows indicate different species formed by RepA proteins corresponding to monomers and dimers, while the position of multimers was marked with brackets.

A time course experiment with a fixed concentration of DMP (10 mM) revealed that each of the tested RepA was initially fixed into covalently bound dimers, but yet the multimeric forms were observed very soon (Fig 7). Three of the tested His_6_-RepA proteins, namely RepA/a, RepA/c, and RepA/d displayed similar kinetics of the respective dimer/multimer formation: they required from 3 to 5 min to form dimers/multimers, while after 15 min the reaction reached a plateau (Fig 7A, 7C and 7D). On the contrary, the RepA/b protein formed dimers rapidly in the solution: after 30 s of incubation with 10 mM of DMP at 28°C; after another 30 s, multimers were visible and after 3 min the reaction reached a plateau (Fig 7B).

**Fig 8:**
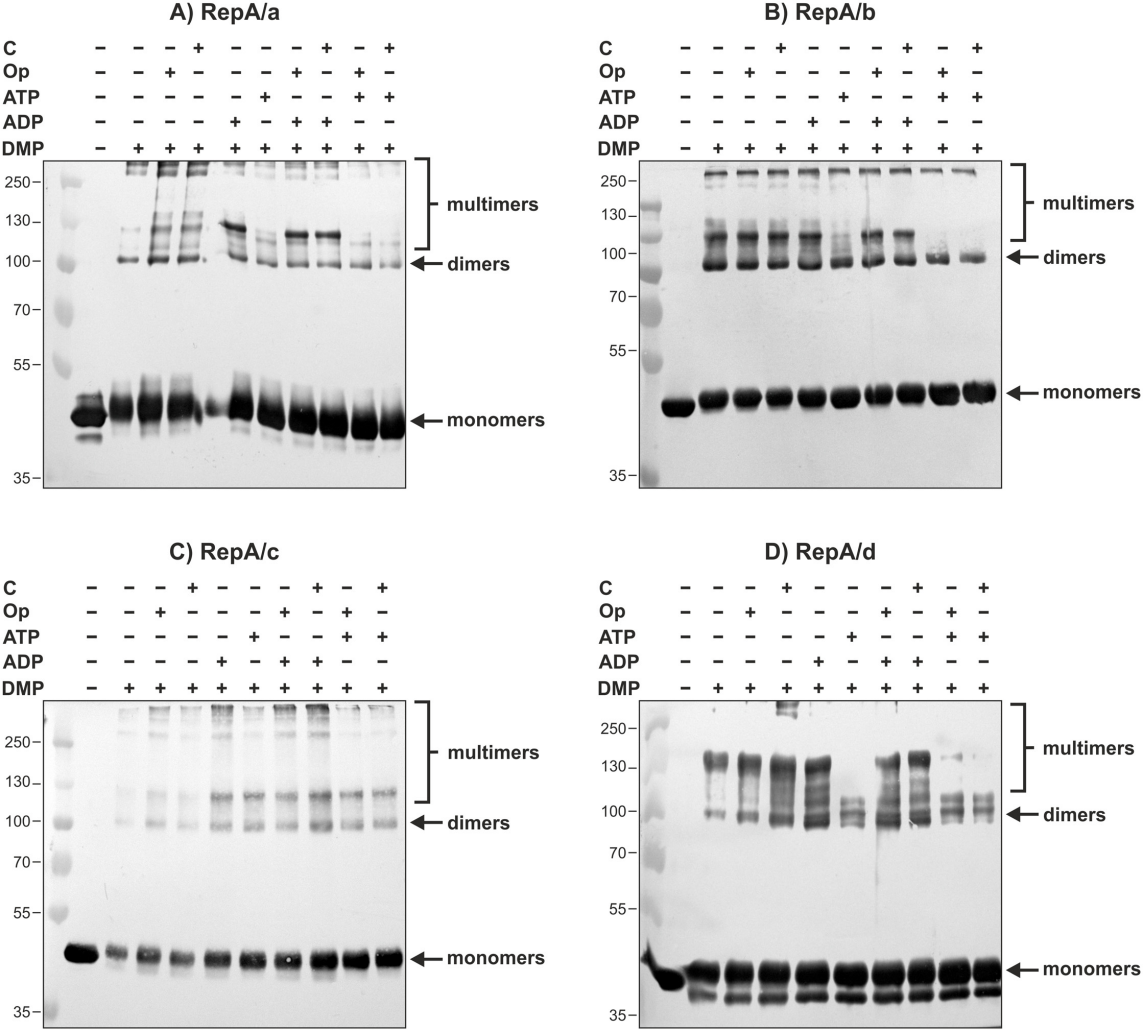
Oligomerization pattern of His_6_-RepA/a-d in the presence of ADP/ATP, specific and nonspecific DNA. Western blots are showing the products of cross-linking of individual His_6_-tagged RepA proteins (0.1–0.2 nM), following 1 h incubation at 28°C with 10 mM DMP and additional compounds. '+' means the presence of: 'C'—nonspecific DNA competitor (10–50 ng); 'Op'—specific operator DNA (10–50 ng); ADP (2 mM) or ATP (2 mM). The thin lines indicate the marker bands whose molecular masses are expressed in kDa. Arrows indicate different species formed by RepA proteins corresponding to monomers and dimers, while the position of multimers was marked with brackets.

To gain further insight into the RepA protein oligomerization pattern, additional components such as ATP, ADP, and specific and non-specific DNA in various combinations were included in the cross-linking reactions (Fig 8). In general, the oligomerization pattern of the His_6_-RepA proteins seemed not to be influenced *in vitro* by the specific or non-specific DNA. On the other hand, when ATP was present in a cross-linking reaction, the bands corresponding to putative multimers were absent or weakly visible for most of the tested RepA and this phenomenon was independent of the kind of tested DNA (Fig 8A, 8B and 8D). In the case of the RepA/c protein, the presence of ATP in the cross-linking reaction did not affect the multimer formation (Fig 8C). However, the addition of ATP slightly diminished the intensity of bands corresponding to high molecular weight multimers (Fig 8C).

These results showed that individual RepA proteins formed dimers and multimers in the solution. The RepA proteins differed slightly in the oligomerization pattern, which was not dependent on the presence of specific or non-specific DNA but was affected by ATP or ADP.

### BACTH analysis of RepA and RepB interplays

**Fig 9:**
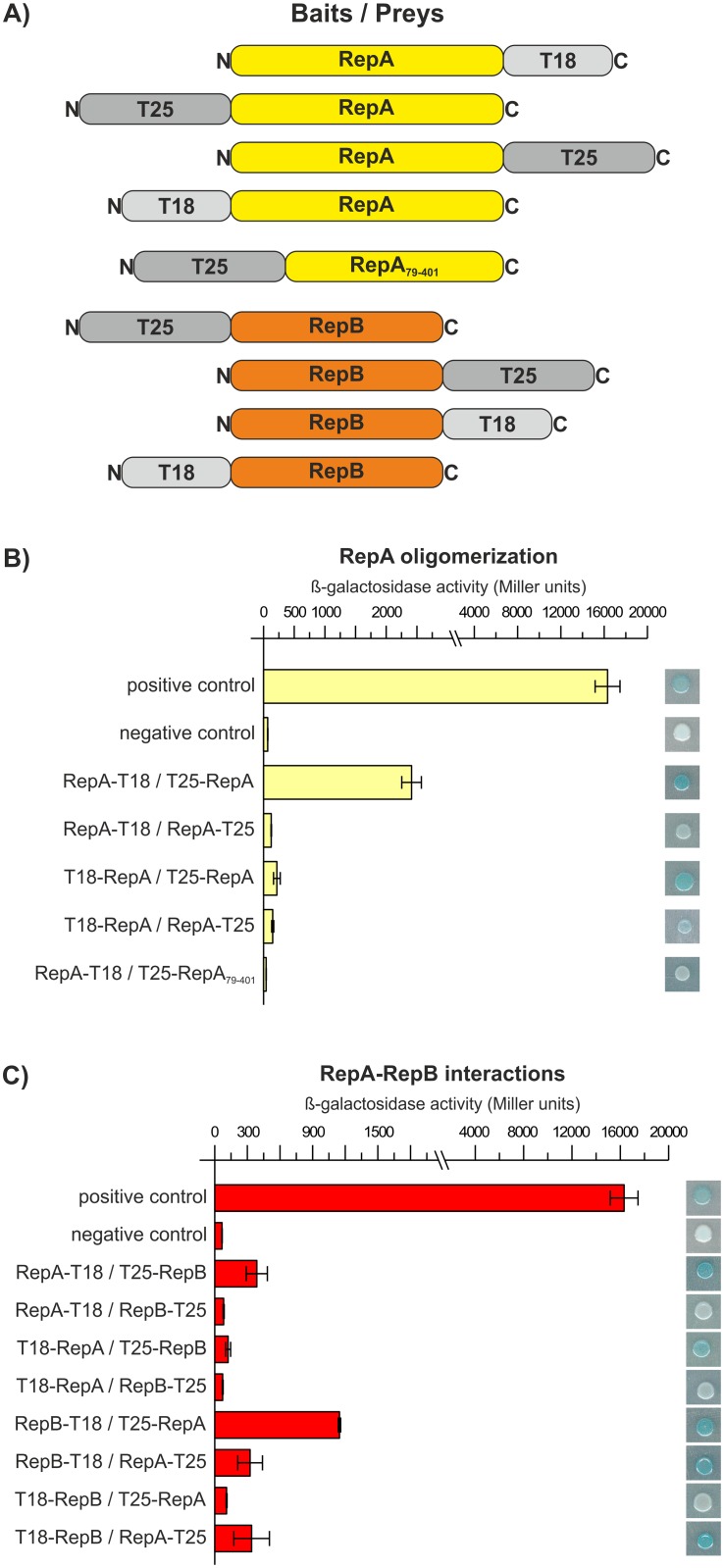
BACTH analysis of interactions of RepA and RepB proteins. (A) The schematic representation of full length RepA and RepB, which were translationally fused to N- and C-terminus of T18 and T25 functional domains of CyaA protein, as well as constructed 'prey' hybrid plasmid which comprised N-terminally truncated RepA fused to T25 domain. (B) RepA/RepA and (C) RepA/RepB interactions in BACTH analysis which were quantified by measuring of β-galactosidase activity (Miller units) in hybrid cotransformants containing bait and prey plasmids. Each value (with standard deviation—extended bars) is the average of at least three independent measurements. Clones of each 'bait'/'prey' plasmid cotransformation were also spotted on LB agar with appropriate antibiotics and X-gal, IPTG, along with positive and negative controls. The colour formation indicates positive clones in which interaction of studied proteins was observed, while the negative clones remain white.

The results presented above demonstrated the self-association ability of RepA as well as the possibility of reciprocal RepA and RepB interaction in the regulation of *repABC* operons. To validate these results, a bacterial two-hybrid system (BACTH) [42] was employed for examination of interplays between RepA and RepB. Since numerous recombinant plasmid constructs are usually required in two-hybrid based studies, only proteins originating from the pRleTA1b plasmid were subjected to the analysis. Both the respective *repA* and *repB* genes were cloned into pUT18, pUT18C, pKT25, and pKNT25 vectors to construct T18- or T25-gene fusions in various combinations (Fig 9A). Then, the *E*. *coli* DHM1 *cya* reporter strain was sequentially transformed with all plasmids expressing fusion proteins. Positive clones with blue colouring and reporter activity substantially higher than in the control, representing the interacting RepA-RepA proteins, were obtained for one of the tested combinations of fusion plasmids RepA-T18/T25-RepA, demonstrating the ability of RepA to form homooligomeric forms *in vivo* (Fig 9B). To map the putative oligomerization domain in the N-terminal part of the RepA protein, a truncated version of RepA comprising 79–401 aa was prepared as a 'prey' and cotransformed with a plasmid expressing RepA as a 'bait' (Fig 9A). No positive blue clones were obtained for this combination of fusion plasmids, in contrast to the high β-galactosidase activity observed for intact RepA-RepA proteins (Fig 9B). Altogether, these results suggested the location of the oligomerization domain in the N-terminus of the RepA protein. In the case of the RepA-RepB interaction, a high level of β-galactosidase activity was obtained in one combination of fusion plasmids (RepB-T18/T25-RepA) (Fig 9C). For several other clones, the reporter activity was higher than in the negative control but substantially lower than in positive clones in which RepA-RepA interaction was observed (Fig 9B and 9C). The results obtained confirm the capability of RepA and RepB proteins of interactions *in vivo*, and the low reporter activities may indicate that such interaction was weak under the experimental conditions applied.

## Discussion

Autoregulation of partition (*par*) operons is a common theme in low-copy number plasmids. However, knowledge about transcriptional regulation is somewhat scarce for most of the rhizobial *repABC* systems and well developed only for two plasmids: *A*. *tumefaciens* pTiR10 and *R*. *etli* p42d [17–19, 21, 43]. The diversity of *repABC* cassettes related to the specific structural and transcriptional regulatory elements may substantially affect the regulation and functioning of particular plasmids in a multipartite genome. Our comprehensive approach focusing on the entire set of plasmids instead of just one led to the observation that the level of transcription of the individual RtTA1 *rep* operons varied: the reporter activity in the *lacZ* fusion comprising promoter of pRleTA1a (pSym) was almost twice as high as that in other RtTA1 *rep* regions. In our previous studies, we showed that horizontal acquisition was the main plausible contributor to the origin of RtTA1 plasmids and pSym could be the newest plasmid of this strain [31]. The presence of putative *tra-trb* genes upstream of pRleTA1a *repABC*, which may be related to the increased transcriptional activity of pRleTA1a *rep* promoter, is another example supporting the diversity of this *rep* region. The *repABC* operon of pTiR10 is functionally linked with *trb-tra* genes and contains four promoters, which respond to diverse environmental signals that modulate its transcription [18, 43]. However, in the pRleTA1a *traI-repA* intergenic sequence, no conserved motifs or segments such as *tra*-boxes were found, which could be indicative of conjugative properties of this plasmid [1, 18]. Similarly, the *R*. *etli* p42d symbiotic plasmid contains a *traI* pseudogene upstream of the *repABC* operon but without *tra* boxes [19], suggesting its inability to detect quorum-sensing signals.

The individual RtTA1 *repABC* operons were negatively regulated by cooperation of RepA and RepB. Only two rhizobial RepA proteins have earlier been shown to mediate autorepression: the RepA of pTiR10 and the RepA of p42d [18, 19]. Both these proteins were recognized as major *cis*-acting autorepressors of respective operons causing substantial reduction of respective promoter activity. In our studies, the constructs expressing solely RepA *in cis* from its own promoter resulted in up to 50% reduction of promoter activity and, in the case of the *repABC*/b operon, RepA alone did not affect the promoter activity. Our data strongly suggest that RepA is important for autorepression but the full repression of individual RtTA1 *repABC* operons is possible only in the presence of both of RepA and RepB, which can be provided *in trans*. This diverse RepA effect on the transcriptional activity of particular RtTA1 *rep* operons further underlines some functional dissimilarity between the individual *repABC* cassettes residing in one cell. Recently, Pérez-Oseguera and Cevallos [21] have shown that RepA alone repressed the transcription of the p42d *repABC* operon only marginally, while both RepA and RepB decreased its activity by approximately 50%.

It was shown for the p42d plasmid that the elements required for full repression of the *repABC* operon comprise RepA, RepB in conjunction with *parS*, and operator sequences [21]. Chai and Winans [17] postulate that RepB increases the affinity of RepA for the binding site at the *repABC* P4 promoter of pTiR10, but also indicate that removal of the RepB binding site (i.e. *parS*) enhances the operon transcription approximately fourfold. Our results allowed us to conclude that presence of the *parS* element is not mandatory to achieve strong repression of the *repABC* operon, whereas RepA-RepB protein interaction seems to be crucial for such regulation. However, since both RepA and RepB are DNA-binding proteins, it is rather hard to define unambiguously the exact nature of the protein–DNA and protein–protein interactions in such a heterogeneous nucleoprotein complex required for *rep* operon regulation. The presented results strongly support the action of RepB as a corepressor and this result stays in agreement with the data provided for p42d [21]. Furthermore, the data discussed above clearly suggest RepA and RepB interplay. By means of BACTH, we have shown that RepA and RepB interact *in vivo*, confirming engagement of these proteins in transcription regulation of the *repABC* operon and presumably (by similarity with ParA/ParB) in plasmid partition.

What is especially important, we have demonstrated highly specific RepA binding to the Op and no cross reactivity between individual RepA and Op from non-parental *repABC* cassettes. Since RepA/RepB are capable of negative regulation of their own transcription and this process plays a crucial role in plasmid incompatibility [21], the specific interaction between RepA and the Op element seems to be one of the fundamental aspects of coexistence of several plasmids equipped with a similar replication system in one bacterial cell.

The DNA binding properties of ParA family members are known to be strongly dependent on whether the protein is bound with ATP or ADP [44]. In the presence of ATP, ParA is capable of binding to non-specific DNA. ParB stimulates the ParA ATPase activity leading to the accumulation of the ParA-ADP repressor form in which ParA binds exclusively to the *parAB* operator [3, 45]. In this regard, all RtTA1 RepA proteins behave similarly to ParA: individual RepA-ADP can bind specifically to their own Op sequence, while the addition of ATP stimulates their non-specific DNA binding. Similar results with respect to the ATP/ADP effect on RepA-Op binding have recently been described for p42d [21]. Oppositely, the RepA binding affinity to the Op sequence overlapping the *repABC* P4 promoter of pTiR10 was equally increased by adenosine di- and triphosphates. The presence of RepB clearly stimulated binding of RepA in both presence and absence of the nucleotide cofactor [18]. The ability of RepA to bind the non-specific DNA implicates its involvement in plasmid partition. A model was presented in which P1 plasmids dynamically associate with the bacterial nucleoid via ParA, positioning plasmids on the bacterial nucleoid, which serves as a matrix support for plasmid partition [3, 46, 47].

Undoubtedly, both ParA activities, i.e. *par* operon regulation and plasmid partition, are related to its ability to dimerize/oligomerize: the ParA filaments can push or pull replicated plasmids apart [48]. In our study, for most of RepA, the dimers were formed independently of ATP or ADP, but the presence of ATP stabilized dimers and/or diminished the amount of oligomers. Similar data have recently been obtained for RepA of p42d [21]. The result is somewhat puzzling in comparison with what is known about ParA. The ParA-ADP structure revealed that ADP binding locks the proteins in a specific dimer state and mediates folding of its C-terminal region important for operator binding [49]. In our studies, the self-association ability of RepA was confirmed *in vivo* using a bacterial two-hybrid system (BACTH). Using this approach, we have also demonstrated that the N-terminal part of RepA/b comprising 78 aa residues is crucial for protein oligomerization. The crystal structure of ParA showed that its N-terminus contained an elongated α-helix, which functions in dimerization, as well as a winged helix-turn-helix DNA binding motif. Such a region is typical for Type Ia NTPases and is not found e.g. in the Ib class [4, 49]. A similar secondary structure was also predicted for the RtTA1 RepA proteins. It has recently been demonstrated that a p42d RepA mutant with a deletion of nine amino acid residues partially overlapping the HTH motif was unable to repress the operon transcription either alone or in the presence of RepB, showing that this region was crucial in DNA binding [21].

A more general conclusion from the data discussed above is that a slightly different mode employed for regulation of particular *rep* cassettes is a result of fine differences related to organization of promoter-operator regions, secondary structures of RepA proteins, and their oligomerization abilities. On the other hand, the several functionally conserved RepA interact in a very specific manner only with parental operons allowing coexistence in one bacterial cell of numerous plasmids possessing similar replication/partition systems. It should be strongly emphasized that all of these functionally important details could come to light by the comprehensive approach focusing on the entire set of plasmids, which we employed.

## Supporting Information

S1 Table Primers used in this study. (DOC)

## References

[1] Cevallos MA, Cervantes-Rivera R, Gutiérrez-Ríos RM. The *repABC* plasmid family. Plasmid. 2008;60(1):19–37. 10.1016/j.plasmid.2008.03.001 . 18433868

[2] Slavcev RA, Funnell BE. Identification and characterization of a novel allele of *Escherichia coli dnaB* helicase that compromises the stability of plasmid P1. J Bacteriol. 2005;187(4):1227–37. 10.1128/JB.187.4.1227-1237.2005 15687186 PMC545633

[3] Vecchiarelli AG, Han YW, Tan X, Mizuuchi M, Ghirlando R, Biertümpfel C, et al ATP control of dynamic P1 ParA-DNA interactions: a key role for the nucleoid in plasmid partition. Mol Microbiol. 2010;78(1):78–91. 10.1111/j.1365-2958.2010.07314.x 20659294 PMC2950902

[4] Schumacher MA. Bacterial plasmid partition machinery: a minimalist approach to survival. Curr Opin Struct Biol. 2012;22(1):72–9. 10.1016/j.sbi.2011.11.001 . 22153351 PMC4824291

[5] Thomas CM. Paradigms of plasmid organization. Mol Microbiol. 2000;37(3):485–91. . 10931342 10.1046/j.1365-2958.2000.02006.x

[6] Bignell C, Thomas CM. The bacterial ParA-ParB partitioning proteins. J Biotechnol. 2001;91(1):1–34. . 11522360 10.1016/s0168-1656(01)00293-0

[7] Campbell CS, Mullins RD. *In vivo* visualization of type II plasmid segregation: bacterial actin filaments pushing plasmids. J Cell Biol. 2007;179(5):1059–66. 10.1083/jcb.200708206 18039937 PMC2099209

[8] Ebersbach G, Gerdes K. Plasmid segregation mechanisms. Annu Rev Genet. 2005;39:453–79. 10.1146/annurev.genet.38.072902.091252 . 16285868

[9] Gerdes K, Møller-Jensen J, Bugge Jensen R. Plasmid and chromosome partitioning: surprises from phylogeny. Mol Microbiol. 2000;37(3):455–66. . 10931339 10.1046/j.1365-2958.2000.01975.x

[10] Ramírez-Romero MA, Soberón N, Pérez-Oseguera A, Téllez-Sosa J, Cevallos MA. Structural elements required for replication and incompatibility of the *Rhizobium etli* symbiotic plasmid. J Bacteriol. 2000;182(11):3117–24. 10809690 10.1128/jb.182.11.3117-3124.2000 PMC94497

[11] Pinto UM, Pappas KM, Winans SC. The ABCs of plasmid replication and segregation. Nat Rev Microbiol. 2012;10(11):755–65. 10.1038/nrmicro2882 . 23070556

[12] Bartosik D, Baj J, Wlodarczyk M. Molecular and functional analysis of pTAV320, a *repABC*-type replicon of the *Paracoccus versutus* composite plasmid pTAV1. Microbiology. 1998;144 (Pt 11):3149–57. . 9846751 10.1099/00221287-144-11-3149

[13] MacLellan SR, Zaheer R, Sartor AL, MacLean AM, Finan TM. Identification of a megaplasmid centromere reveals genetic structural diversity within the *repABC* family of basic replicons. Mol Microbiol. 2006;59(5):1559–75. 10.1111/j.1365-2958.2006.05040.x . 16468995

[14] Pappas KM. Cell-cell signaling and the *Agrobacterium tumefaciens* Ti plasmid copy number fluctuations. Plasmid. 2008;60(2):89–107. 10.1016/j.plasmid.2008.05.003 . 18664372

[15] Castillo-Ramírez S, Vázquez-Castellanos JF, González V, Cevallos MA. Horizontal gene transfer and diverse functional constrains within a common replication-partitioning system in Alphaproteobacteria: the *repABC* operon. BMC Genomics. 2009;10:536 10.1186/1471-2164-10-536 19919719 PMC2783167

[16] Bartosik D, Szymanik M, Wysocka E. Identification of the partitioning site within the *repABC*-type replicon of the composite *Paracoccus versutus* plasmid pTAV1. J Bacteriol. 2001;183(21):6234–43. 10.1128/JB.183.21.6234-6243.2001 11591666 PMC100104

[17] Chai Y, Winans SC. RepB protein of an *Agrobacterium tumefaciens* Ti plasmid binds to two adjacent sites between *repA* and *repB* for plasmid partitioning and autorepression. Mol Microbiol. 2005;58(4):1114–29. 10.1111/j.1365-2958.2005.04886.x . 16262794

[18] Pappas KM, Winans SC. The RepA and RepB autorepressors and TraR play opposing roles in the regulation of a Ti plasmid *repABC* operon. Mol Microbiol. 2003;49(2):441–55. . 12828641 10.1046/j.1365-2958.2003.03560.x

[19] Ramírez-Romero MA, Téllez-Sosa J, Barrios H, Pérez-Oseguera A, Rosas V, Cevallos MA. RepA negatively autoregulates the transcription of the *repABC* operon of the *Rhizobium etli* symbiotic plasmid basic replicon. Mol Microbiol. 2001;42(1):195–204. . 11679078 10.1046/j.1365-2958.2001.02621.x

[20] Soberón N, Venkova-Canova T, Ramírez-Romero MA, Téllez-Sosa J, Cevallos MA. Incompatibility and the partitioning site of the *repABC* basic replicon of the symbiotic plasmid from *Rhizobium etli* . Plasmid. 2004;51(3):203–16. 10.1016/j.plasmid.2004.01.005 . 15109827

[21] Pérez-Oseguera A, Cevallos MA. RepA and RepB exert plasmid incompatibility repressing the transcription of the *repABC* operon. Plasmid. 2013;70(3):362–76. 10.1016/j.plasmid.2013.08.001 . 24016735

[22] Venkova-Canova T, Soberón NE, Ramírez-Romero MA, Cevallos MA. Two discrete elements are required for the replication of a *repABC* plasmid: an antisense RNA and a stem-loop structure. Mol Microbiol. 2004;54(5):1431–44. 10.1111/j.1365-2958.2004.04366.x . 15554980

[23] Chai Y, Winans SC. A small antisense RNA downregulates expression of an essential replicase protein of an *Agrobacterium tumefaciens* Ti plasmid. Mol Microbiol. 2005;56(6):1574–85. 10.1111/j.1365-2958.2005.04636.x . 15916607

[24] MacLellan SR, Smallbone LA, Sibley CD, Finan TM. The expression of a novel antisense gene mediates incompatibility within the large *repABC* family of alpha-proteobacterial plasmids. Mol Microbiol. 2005;55(2):611–23. 10.1111/j.1365-2958.2004.04412.x . 15659174

[25] Cervantes-Rivera R, Romero-López C, Berzal-Herranz A, Cevallos MA. Analysis of the mechanism of action of the antisense RNA that controls the replication of the *repABC* plasmid p42d. J Bacteriol. 2010;192(13):3268–78. 10.1128/JB.00118-10 20435728 PMC2897686

[26] Rivera-Urbalejo A, Pérez-Oseguera A, Carreón-Rodríguez OE, Cevallos MA. Mutations in an antisense RNA, involved in the replication control of a *repABC* plasmid, that disrupt plasmid incompatibility and mediate plasmid speciation. Plasmid. 2015;78:48–58. 10.1016/j.plasmid.2015.01.004 . 25644116

[27] Yip CB, Ding H, Hynes MF. Counter-transcribed RNAs of *Rhizobium leguminosarum repABC* plasmids exert incompatibility effects only when highly expressed. Plasmid. 2015;78:37–47. 10.1016/j.plasmid.2014.12.003 . 25530178

[28] Cervantes-Rivera R, Pedraza-López F, Pérez-Segura G, Cevallos MA. The replication origin of a *repABC* plasmid. BMC Microbiol. 2011;11:158 10.1186/1471-2180-11-158 21718544 PMC3155836

[29] Pinto UM, Flores-Mireles AL, Costa ED, Winans SC. RepC protein of the octopine-type Ti plasmid binds to the probable origin of replication within *repC* and functions only *in cis* . Mol Microbiol. 2011;81(6):1593–606. 10.1111/j.1365-2958.2011.07789.x . 21883520

[30] Król JE, Mazur A, Marczak M, Skorupska A. Syntenic arrangements of the surface polysaccharide biosynthesis genes in *Rhizobium leguminosarum* . Genomics. 2007;89(2):237–47. 10.1016/j.ygeno.2006.08.015 . 17014983

[31] Mazur A, Majewska B, Stasiak G, Wielbo J, Skorupska A. *repABC*-based replication systems of *Rhizobium leguminosarum* bv. *trifolii* TA1 plasmids: incompatibility and evolutionary analyses. Plasmid. 2011;66(2):53–66. 10.1016/j.plasmid.2011.04.002 . 21620885

[32] Sambrook J, Fritsch EF, Maniatis T. Molecular cloning: a laboratory manual 2nd ed. Cold Spring Harbor, New York: Cold Spring Harbor Laboratory Press; 1989.

[33] Vincent JM. A manual for the practical study of root-nodule bacteria. Oxford: Blackwell Scientific; 1970.

[34] Spaink HP, Okker RJ, Wijffelman CA, Pees E, Lugtenberg BJ. Promoters in the nodulation region of the *Rhizobium leguminosarum* Sym plasmid pRL1JI. Plant Mol Biol. 1987;9(1):27–39. 10.1007/BF00017984 . 24276795

[35] Kovach ME, Elzer PH, Hill DS, Robertson GT, Farris MA, Roop RM 2nd, et al Four new derivatives of the broad-host-range cloning vector pBBR1MCS, carrying different antibiotic-resistance cassettes. Gene. 1995;166(1):175–6. . 8529885 10.1016/0378-1119(95)00584-1

[36] Brown CM, Dilworth MJ. Ammonia assimilation by rhizobium cultures and bacteroids. J Gen Microbiol. 1975;86(1):39–48. . 234505 10.1099/00221287-86-1-39

[37] Miller JH. Experiments in molecular genetics Cold Spring Harbor, New York: Cold Spring Harbor Laboratory Press; 1972.

[38] Reese MG. Application of a time-delay neural network to promoter annotation in the *Drosophila melanogaster* genome. Comput Chem. 2001;26(1):51–6. . 11765852 10.1016/s0097-8485(01)00099-7

[39] Knudsen S. Promoter2.0: for the recognition of PolII promoter sequences. Bioinformatics. 1999;15(5):356–61. . 10366655 10.1093/bioinformatics/15.5.356

[40] Jones DT. Protein secondary structure prediction based on position-specific scoring matrices. J Mol Biol. 1999;292(2):195–202. 10.1006/jmbi.1999.3091 . 10493868

[41] Narasimhan G, Bu C, Gao Y, Wang X, Xu N, Mathee K. Mining protein sequences for motifs. J Comput Biol. 2002;9(5):707–20. 10.1089/106652702761034145 . 12487759

[42] Karimova G, Pidoux J, Ullmann A, Ladant D. A bacterial two-hybrid system based on a reconstituted signal transduction pathway. Proc Natl Acad Sci U S A. 1998;95(10):5752–6. 9576956 10.1073/pnas.95.10.5752 PMC20451

[43] Pappas KM, Winans SC. A LuxR-type regulator from *Agrobacterium tumefaciens* elevates Ti plasmid copy number by activating transcription of plasmid replication genes. Mol Microbiol. 2003;48(4):1059–73. . 12753196 10.1046/j.1365-2958.2003.03488.x

[44] Davey MJ, Funnell BE. The P1 plasmid partition protein ParA. A role for ATP in site-specific DNA binding. J Biol Chem. 1994;269(47):29908–13. . 7961987

[45] Castaing JP, Bouet JY, Lane D. F plasmid partition depends on interaction of SopA with non-specific DNA. Mol Microbiol. 2008;70(4):1000–11. 10.1111/j.1365-2958.2008.06465.x . 18826408

[46] Erdmann N, Petroff T, Funnell BE. Intracellular localization of P1 ParB protein depends on ParA and *parS* . Proc Natl Acad Sci U S A. 1999;96(26):14905–10. 10611311 10.1073/pnas.96.26.14905 PMC24746

[47] Sengupta M, Nielsen HJ, Youngren B, Austin S. P1 plasmid segregation: accurate redistribution by dynamic plasmid pairing and separation. J Bacteriol. 2010;192(5):1175–83. 10.1128/JB.01245-09 19897644 PMC2820840

[48] Howard M, Gerdes K. What is the mechanism of ParA-mediated DNA movement? Mol Microbiol. 2010;78(1):9–12. 10.1111/j.1365-2958.2010.07316.x . 20659290

[49] Dunham TD, Xu W, Funnell BE, Schumacher MA. Structural basis for ADP-mediated transcriptional regulation by P1 and P7 ParA. EMBO J. 2009;28(12):1792–802. 10.1038/emboj.2009.120 19461582 PMC2699355

[50] Chakravorty AK, Zurkowski W, Shine J, Rolfe BG. Symbiotic nitrogen fixation: molecular cloning of *Rhizobium* genes involved in exopolysaccharide synthesis and effective nodulation. J Mol Appl Genet. 1982;1(6):585–96. . 6296257

[51] Rosenberg C, Huguet T. The pAtC58 plasmid of *Agrobacterium tumefaciens* is not essential for tumour induction. Mol Gen Genet. 1984;196:533–6.

