# Supplementary material for: Plasmid-Encoded RepA Proteins Specifically Autorepress Individual repABC Operons in the Multipartite Rhizobium leguminosarum bv. trifolii Genome
Source: PLoS One. 2015 Jul 6;10(7):e0131907. doi: 10.1371/journal.pone.0131907 (PMC4492784; doi:10.1371/journal.pone.0131907)
Supplement: S1 Table — (DOC) [file pone.0131907.s001.doc]

**S1 Table. Primers used in this study.**

| **Primer name** | **Primer sequence (5'-3')**^a^ |
| --- | --- |
| Primers used for *lacZ* transcriptional fusions | |
| *repABC* of pRleTA1a | |
| Prepa-1FwKpn | aaaggtaccCCGGTACATTTGATCGAGAAGTGCTG |
| Prepa-3RwXba | aaatctagaAAGGTGCGCAACGATTTCA |
| PrepaFw2Kpn | aaaggtaccGTTGCCAGCAGCGGTTCATTCGTT |
| Prepa-3FwKpn | aaaggtaccAACCCTTGCGACGGAAAATACG |
| Prepa-2RwXba | aaatctagaCGGTCGGACTGTCACGGTAGG |
| Prepa-4RwXba | aaatctagaCCTACCCCATCGGCGTCTCT |
| Prepa-1RwXba | aaatctagaACAATGGCTTCAGAACCGCTCAAT |
| *repABC* of pRleTA1b | |
| Prepb-4FwKpn | aaaggtaccATTGATCGCCTGTATGGTGAGA |
| Prepb-3RwXba | aaatctagaCTTGTCGCGGAGGAGCTTCAGTTG |
| Prepb-3FwKpn | aaaggtaccCGGCAGCTGAGCCCTATTGTTG |
| Prepb-5FwKpn | aaaggtaccTTGACGCTATGTTGAAAGAAAAG |
| Prepb-5RwXba | aaatctagaTGCGGTCCCTTGCCATCCAG |
| Prepb-2RwXba | aaatctagaTGGCCTCGGCAATCTCGTTTTT |
| Prepb-4RwXba | aaatctagaTTTCTTTTGCGGCCTACTCTCCTG |
| Prepb-1RwXba | aaatctagaAGGCGATCGCGTCTGCTC |
| *repABC* of pRleTA1c | |
| c-ABFw1 | aaaggtaccGTGGGCTGGAAAACGAAAACTTGTA |
| Prepc-5RwXba | aaatctagaGCTGAGCATCGTCGAGTCCTGTG |
| c-ABFw2 | aaaggtaccCGTCGAATCCGGTTTGTTACCAC |
| Prepc-4FwKpn | aaaggtaccCATTTGCGCAAAATGACCCG |
| Prepc-6RwXba | aaatctagaACCGGCGGCAGTCTTTTC |
| Prepc-3RwXba | aaatctagaGGGCCAGTTCGTTCTTCGGATAAA |
| c-ABrev1 | aaatctagaGCGCGTTAACTCCTATCCGAATTG |
| c-ABrev2 | aaatctagaGACAACCGGTCTAATTGCCTCAT |
| Prepc-4RwXba | aaatctagaCTTGGCGGCGGCGGCTTCTT |
| *repABC* of pRleTA1d | |
| Prepd-1FwKpn | aaaggtaccAGGTTCCGACGGGCTTCTGGTGA |
| Prepd-3RwXba | aaatctagaGCGGCTACCTGGCGGAGATAA |
| PrepdFw2Kpn | aaaggtaccACTGACGGTTTGGTTAAGCATTTG |
| Prepd-3FwKpn | aaaggtaccGCATATAGTAACTGCGCAACTGAAGC |
| Prepd-4FwKpn | aaaggtaccTCGTTCAAAATTGCGCAGTTACCT |
| Prepd-5FwKpn | aaaggtaccGGAAAAAGATCATGCAGCCGAG |
| Prepd-2RwXba | aaatctagaGCGCTTTCCATTCGGGCTGTC |
| Prepd-4RwXba | aaatctagaGCGTCGTTGGGGGCCTTTTTCT |
| Prepd-1RwXba | aaatctagaGCTCACTCGCCCGCTCCATCTT |
| Primers used for constructs in expression plasmid vectors | |
| repA-aFwBam | aaaggatccGATCAGATGACGACACGCAAACC |
| repA-aRwKpn | aaaggtaccTCATGACCGTCCCCATACCGAT |
| repA-bFwBam | aaaggatccGGTGTTTTGGACAACATTAGCGTA |
| repA-bRwHind | aaaaagcttTCATGcTCGACCCCAGGC |
| repA-cFwBam | aaaggatccCCGACCAAGGCCGCGATTG |
| repA-cRwHind | aaaaagcttTCATGTCCTGCTCCATGCGCT |
| repA-dFwBam | aaaggatccCAGCCGAGTCTTGCTCTTCAA |
| repA-dRwHind | aaaaagcttCTATTTCCTTCCCCAGGTCGA |
| repBaFw | aaaggatccACGAATGCCAAAGAACGCTCCA |
| repBaRw | aaaaagcttCTACCCCATCGGCGTCTCTCC |
| repBbFw | aaaggatccAGCAGACGCGATCGCCTGAAA |
| repBbRw | aaaaagcttCTACTCTCCTGCTTGCAACTTGCC |
| repBcFw | aaaggatccAGCCCAAAAGGACGCGACATC |
| repBcRw | aaaaagcttTTTTCCTTCATTTTCCTCTTCAAACTCG |
| repBdFw | aaaggatccGCGCGCAAGAACCTCATCG |
| repBdRw | aaaaagcttCTACGCTCCTCTGTCCTTGTTGAA |
| Primers used for EMSA assay | |
| *repABC* of pRleTA1a | |
| PrepaFw2Kpn | aaaggtaccGTTGCCAGCAGCGGTTCATTCGTT |
| Prepa-3RwXba | aaatctagaAAGGTGCGCAACGATTTCA |
| Prepa-3FwKpn | aaaggtaccAACCCTTGCGACGGAAAATACG |
| Ptrb/aF | aaagaattcCCTCGCGACGGTTTTTCACTG |
| repA-aFwBam | aaaggatccGATCAGATGACGACACGCAAACC |
| *repABC* of pRleTA1b | |
| Prepb-4FwKpn | aaaggtaccATTGATCGCCTGTATGGTGAGA |
| Prepb-3RwXba | aaatctagaCTTGTCGCGGAGGAGCTTCAGTTG |
| Prepb-3FwKpn | aaaggtaccCGGCAGCTGAGCCCTATTGTTG |
| Prepb-5FwKpn | aaaggtaccTTGACGCTATGTTGAAAGAAAAG |
| repA-bFwBam | aaaggatccGGTGTTTTGGACAACATTAGCGTA |
| *repABC* of pRleTA1c | |
| c-ABFw1 | aaaggtaccGTGGGCTGGAAAACGAAAACTTGTA |
| Prepc-5RwXba | aaatctagaGCTGAGCATCGTCGAGTCCTGTG |
| c-ABFw2 | aaaggtaccCGTCGAATCCGGTTTGTTACCAC |
| Prepc-4FwKpn | aaaggtaccCATTTGCGCAAAATGACCCG |
| repA-cFwBam | aaaggatccCCGACCAAGGCCGCGATTG |
| *repABC* of pRleTA1d | |
| Prepd-1FwKpn | aaaggtaccAGGTTCCGACGGGCTTCTGGTGA |
| Prepd-3RwXba | aaatctagaGCGGCTACCTGGCGGAGATAA |
| PrepdFw2Kpn | aaaggtaccACTGACGGTTTGGTTAAGCATTTG |
| Prepd-3FwKpn | aaaggtaccGCATATAGTAACTGCGCAACTGAAGC |
| Prepd-4FwKpn | aaaggtaccTCGTTCAAAATTGCGCAGTTACCT |
| repA-dFwBam | aaaggatccCAGCCGAGTCTTGCTCTTCAA |
| non-specific competitor | |
| KanRTFw | GGGCGCCCGGTTCTTTTTGTCA |
| KanRTRw | GCCAGTCCCTTCCCGCTTCAGTG |
| Primers used for construction of pMPK plasmid vector (pMP220 derivative) | |
| KanFwKpn | aaaggtaccGGGAAAACGCAAGCGCAAAGAGAA |
| KanRwEco | aaagaattcTCATAGAAGGCGGCGGTGGAATC |
| Primers used for BACTH assay | |
| T25repA-bFwXba | aaatctagaGGGTGTTTTGGACAACATTAGCGTA |
| T18repA-bRwKpn | aaaggtaccGCTCGACCCCAGGCTTTG |
| NT25repA-bRwKpn | aaaggtaccCATGCTCGACCCCAGGC |
| T25repA-bRwKpn | aaaggtaccTCATGCTCGACCCCAGGC |
| T25repAbFw79XHTHdelXba | aaatctagaGAATGGCCGGCGTTCCTACAC |
| T25repB-bFwXba | aaatctagaaAGCAGACGCGATCGCCTGAAA |
| T18repBbKpn | aaaggtaccTCTCCTGCTTGCAACTTGCC |
| T25repB-bRwKpn | aaaggtaccCTACTCTCCTGCTTGCAACTTGCC |
| NT25repB-bRwKpn | aaaggtaccTCTCCTGCTTGCAACTTGCC |

^a^ Introduced restriction sites are underlined
